# Molecular Fingerprint Detection Using Raman and Infrared Spectroscopy Technologies for Cancer Detection: A Progress Review

**DOI:** 10.3390/bios13050557

**Published:** 2023-05-18

**Authors:** Shuyan Zhang, Yi Qi, Sonia Peng Hwee Tan, Renzhe Bi, Malini Olivo

**Affiliations:** 1 Institute of Materials Research and Engineering (IMRE), Agency for Science, Technology and Research (A*STAR), 31 Biopolis Way, Nanos #07-01, Singapore 138634, Singapore; 2 Department of Biomedical Engineering, National University of Singapore (NUS), 4 Engineering Drive 3 Block 4, #04-08, Singapore 117583, Singapore

**Keywords:** biosensor, molecular fingerprint, vibrational spectroscopy, Raman spectroscopy, infrared spectroscopy, biomedical, cancer diagnosis, biomarker

## Abstract

Molecular vibrations play a crucial role in physical chemistry and biochemistry, and Raman and infrared spectroscopy are the two most used techniques for vibrational spectroscopy. These techniques provide unique fingerprints of the molecules in a sample, which can be used to identify the chemical bonds, functional groups, and structures of the molecules. In this review article, recent research and development activities for molecular fingerprint detection using Raman and infrared spectroscopy are discussed, with a focus on identifying specific biomolecules and studying the chemical composition of biological samples for cancer diagnosis applications. The working principle and instrumentation of each technique are also discussed for a better understanding of the analytical versatility of vibrational spectroscopy. Raman spectroscopy is an invaluable tool for studying molecules and their interactions, and its use is likely to continue to grow in the future. Research has demonstrated that Raman spectroscopy is capable of accurately diagnosing various types of cancer, making it a valuable alternative to traditional diagnostic methods such as endoscopy. Infrared spectroscopy can provide complementary information to Raman spectroscopy and detect a wide range of biomolecules at low concentrations, even in complex biological samples. The article concludes with a comparison of the techniques and insights into future directions.

## 1. Introduction

Molecular vibrations, known as “molecular fingerprints,” are crucial processes in physical chemistry and biochemistry. Studying these fingerprints can provide valuable insights into disease mechanisms by tracking the structural changes that occur in biological samples during disease development or treatment. Currently, there is a great clinical interest in developing a rapid and non-invasive methodology that can enable real-time monitoring of the morphological and biochemical modifications occurring in tissues during carcinogenesis, overcoming the limitations of standard biomedical techniques.

Raman and infrared spectroscopies are two extensively utilized vibrational spectroscopy methods for detecting molecular fingerprints. Raman spectroscopy is based on the inelastic scattering of light, which causes molecules to vibrate and emit light of a different frequency than the incident light, and is widely used in chemistry, biology, and materials science for molecular structure and interaction analysis [1,2,3,4,5,6,7,8,9,10,11]. Infrared spectroscopy, on the other hand, uses infrared light to measure the vibrational and rotational motions of molecules in a sample, and is sensitive to different types of functional groups than Raman spectroscopy is.

Combining Raman and infrared spectroscopy can provide complementary information to give a complete characterization of the molecular fingerprints of a sample. Additionally, surface-enhanced techniques such as surface-enhanced Raman spectroscopy (SERS) and surface-enhanced infrared absorption spectroscopy (SEIRA) can detect a wide range of biomolecules at low concentrations, even in complex biological samples. However, the setup of proper sample handling, measurement, and data processing protocols for reproducibly detecting specific disease patterns by surface-enhanced methods is challenging.

There has been extensive research in this field, such as, for example, medical imaging and cancer diagnosis based on Fourier transform infrared spectroscopy techniques [2,3,12], hyperspectral imaging techniques [4,6,7], SEIRA techniques [9,10], and Raman spectroscopy techniques [11,13,14,15,16]. In this review article, we will focus on recent advancements in molecular fingerprint detection using Raman and infrared spectroscopy for cancer detection in the past 10 years. Specifically, we will highlight the identification of specific biomolecules, such as proteins, lipids, and nucleic acids, as well as the examination of the chemical composition of biological samples, including tissues, cells, and biofluids, for various cancer diagnosis applications. We will also review the different Raman and infrared spectroscopy techniques that have been developed and their working principles and instrumentation for better understanding of the analytical versatility of vibrational spectroscopy. Lastly, we will compare these techniques and provide insights into future directions.

## 2. Technology Review

### 2.1. Raman Spectroscopy

In 1928, the Raman effect was first discovered by Chandrashekhara Venkata Raman. This phenomenon, also known as Raman scattering, occurs when photons scatter inelastically, resulting in a change in the wavelength of the incident light beam. The change in wavelength offers insights into the chemical structure of the molecules by revealing their vibrational modes. Raman spectroscopy is a type of vibrational spectroscopy that is used to investigate the low-frequency modes, rotational modes, and vibrational modes of a system. It utilizes the inelastic scattering of monochromatic light, typically from a visible (VIS), near-infrared (NIR), or near-ultraviolet (UV) laser [17]. When a photon of light interacts with a molecule, the majority of the photons are elastically scattered, known as Rayleigh scattering. However, a small fraction of the photons (approximately 1 in 10 million) is scattered inelastically, resulting in a change in energy of the scattered photons. This energy shift is the Raman effect, and the resulting inelastically scattered light is analyzed to give information about the molecular vibrational modes, thus providing a chemical structure fingerprint.

**Figure 1:**
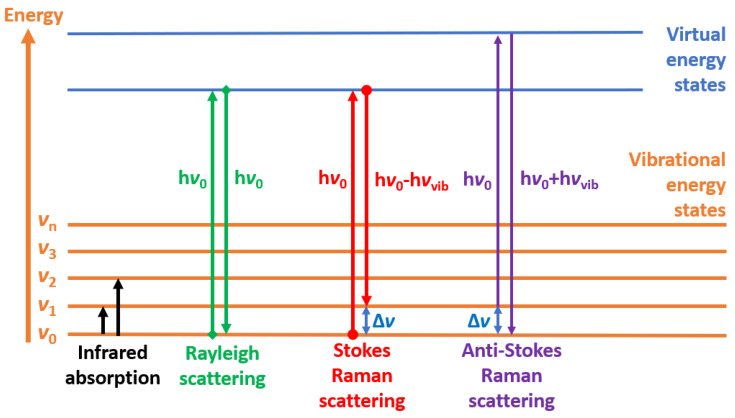
Three types of scattering and their change in energy state due to the monochromatic light excitation of a molecular.

Figure 1 illustrates that when a molecule is subjected to a monochromatic light beam, it can undergo three types of scattering: Rayleigh scattering, Stokes Raman scattering, and anti-Stokes Raman scattering. From the quantum mechanical principle, the vibrational energy states of a diatomic molecule is

(1) $$E_{v}=hv(n+\frac{1}{2})$$

where v is the vibrational frequency, h is Plank’s constant, and n is the vibrational quantum number with integer values. The light from Rayleigh scattering has no change in the vibrational energy level, so the frequency of the scattered light is the same as the incident excitation light. Therefore, although the Rayleigh scattering contains the largest amount of energy of the incident light, it cannot be used for the identification of the molecule. During Stokes Raman scattering, the molecule gains vibrational energy due to the interaction with the incident light, resulting in a decrease in frequency of the scattered light photon. On the other hand, during anti-Stokes Raman scattering, the final energy state of the molecule is lower than the initial state, resulting in an increase in frequency of the scattered light compared to the incident light. Furthermore, Raman scattering is also different from infrared absorption, where the difference between the initial and final energy states is equal to the absorbed photon energy.

**Figure 2:**
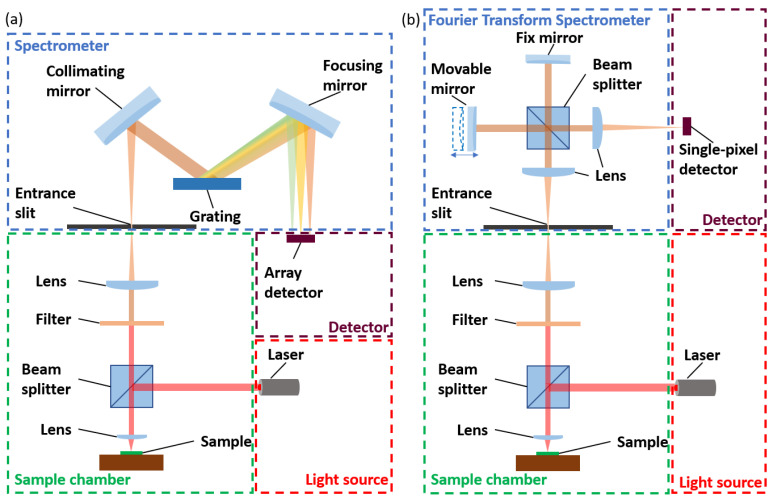
Two approaches for Raman spectroscopy: (**a**) Dispersive spectroscopy and (**b**) Fourier transform (FT) spectroscopy.

A Raman spectrometer is a device that uses the Raman effect to measure the vibrational, rotational, and other low-frequency modes in a system. It consists of four main components: a light source, a sample chamber, a spectrometer, and a detector. The light source is used to excite the sample molecules, causing them to vibrate and emit photons of different energies. The laser source provides a narrow, monochromatic light beam with a single wavelength. This makes them ideal for obtaining high-resolution spectra. Common laser wavelengths used in Raman spectroscopy range from the UV to the NIR region, such as the argon ion laser in 480.0 nm and 514.5 nm, the krypton ion laser in 530.9 nm and 647.1 nm, the He:Ne laser in 632.8 nm, the laser diode in 630 nm and 785 nm, and the Nd:YAG laser in 532 nm and 1064 nm [18,19]. Wavelength in the VIS range (400–700 nm) is the most common because the detector can be a low-cost Si-based array sensor [20,21]. However, the fluorescent effect on the organic subject will influence the signal-to-noise ratio (SNR) of the Raman spectrometer. Thus, there is various emerging research in fluorescence-free Raman spectroscopy with an NIR light source [17]. The sample chamber is used to contain the sample and keep it in a stable environment. The spectrometer is used to separate the different photons according to their energies. Two approaches are the most commonly used: dispersive spectroscopy and Fourier transform (FT) spectroscopy, as shown in Figure 2. Various types of Raman spectroscopy have been developed based on these two approaches. 

Resonance Raman spectroscopy (RRS) uses a laser with a frequency close to the electronic transition energy of the object. The resonance between the incident photon and the analyte enhances the intensity of the Raman scattering. Unlike general Raman scattering, which only occurs at the virtual energy state, resonance Raman can detect the excited electronic state, hence improving the sensitivity of the spectroscopy. RSS has been utilized to explore various aspects of protein structure and dynamics and to investigate conformational changes that occur in proteins, as well as interactions between proteins and other molecules [22]. RRS has also been employed for biomedical applications, such as detecting blood cells and diagnosing skin conditions in vivo [23,24].

A confocal Raman spectrometer (CRS) is a versatile instrument capable of providing a variety of spectroscopic information. CRS provides additional spatial resolution at the micron level by incorporating a confocal optical setup to suppress stray light and realize depth profiling, which can be used to measure multi-layered samples non-invasively. CRS is a powerful tool for testing plastics, polymers, and drugs because the confocal laser can penetrate the surface coating of samples. However, the high power of the laser (>100 mW) may lead to the injury of organic tissues. Fortunately, with the development of semiconductor technology and the improvement of detector performance, many CRS with low-power laser sources (<20 mW) have been investigated [25,26,27] and widely used for the analysis of the stratum corneum, natural moisturizing factor, water content, and other morphology diagnoses of in vivo skin [28].

Spatially offset Raman spectroscopy (SORS) can probe deeper into samples than conventional Raman spectroscopy. SORS has the capability to eliminate scattering photons from the surface of an object by spatially offsetting the laser excitation and scattering collection regions. Hence, the diffuse scattering from the deeper layer tissue of the sample can be detected [29]. In contrast, conventional Raman spectroscopy generally only works on the surface of a sample, while CRS can penetrate 100–200 µm into a transparent or semi-transparent sample. However, SORS can penetrate >2 mm into diffusely scattering samples, making it useful for the non-invasive diagnosis of deep tissue diseases such as cancer monitoring. As a result, SORS has been widely used in biomedical research [29].

Surface-enhanced Raman spectroscopy (SERS) concentrates electromagnetic (EM) energy by metallic nanostructures with surface plasmon optical modes. SERS enhances the intensity of Raman scattering by the combined effect of EM and chemical mechanisms, which improves the level of Raman scattering by 10^8^ to 10^11^ magnitude. When the excitation laser irradiates the metallic nanostructure, the conductive electrons will be delocalized and oscillated. While the excitation laser frequency is resonant with the electron oscillation, localized surface plasmon resonance (LSPR) can enhance the EM field near the metal nanostructure interface, resulting in surface-enhanced Raman scattering [30,31]. SERS can detect single molecules due to the enhancement of Raman scattering. This makes it a useful tool for detecting low-abundance biomolecules such as proteins in bodily fluids, which are important biomarkers for early cancer diagnosis [32,33,34,35]. A SERS-based microfluidic chip has been developed for multiplex protein biomarkers detection [28].

The SERS technique has numerous advantages in cancer diagnosis [36] and therapy due to its high Raman scattered light signal strength. SERS has gained significant popularity in biomedical research over the past decades, as it offers high sensitivity and multiplexing capabilities that are attractive in molecular diagnostics [37]. Applications of SERS in cancer detection include using immunoassays and the detection of biomarkers, single-nucleotide polymorphisms, and circulating tumor cells [32,33,38]. SERS has also been explored as a dynamic technology for point-of-care (POC) monitoring due to its high sensitivity and multiplexing capabilities, making it a promising candidate for rapid diagnostic testing [39]. Despite its high sensitivity and multiplexing capabilities, SERS still faces challenges, such as unreliable and inconsistent signals, a lack of methodologies that can be applied to multiple types of biomarkers, and the expensive cost of portable Raman readers. Nevertheless, promising solutions for SERS in point-of-care (POC) monitoring are being explored, such as the use of more specific and robust capture ligands such as DNA aptamer-coated nanomaterials [40]. SERS has also found applications in cancer imaging and as a tool for targeted drug delivery and photothermal therapy. Recent technical improvements in SERS, including integration into microfluidic chips and hollow crystal photonic fibers, have been highlighted by Vendrell et al. They provide an overview of label-free SERS methodologies and their potential for non-invasive cancer diagnosis and profiling [41]. Wei et al. developed a SERS substrate by utilizing a two-step method known as the noble metal-assisted chemical etching and reduction method, which involves synthesizing gold nanoparticles/silicon nanowire arrays (Au/SiNWA). They then measured the SERS spectra of serum samples from both healthy individuals and gastric cancer patients using the Au/SiNWA substrate [42].

SERS nanoparticles are currently being developed for biomedical purposes, including tumor imaging-guided theranostics and biosensing [43]. Li et al. described the fabrication of SERS nanoparticles that are biocompatible, have high SERS enhancement, and are encapsulated to maintain their Raman fingerprint [44]. The strategy for in vivo SERS imaging involves attaching Raman molecule probes to SERS nanoparticles and covering the SERS-active cores with protective shell materials. The aim is to combine the diagnostic imaging and therapeutic functions of SERS nanoparticles. Qian et al. compared the in vitro cellular binding and in vivo distribution of three types of control SERS nanotags, including plain PEG-coated nanoparticles, IgG1-labeled SERS tags, and smaller nonspecific protein-labeled nanoparticles, and found that the EGFR-targeted nanoparticles were the most efficient for tumor uptake [43]. Recent research in SERS-guided theranostic nanoplatforms has shown promising results in photodynamic therapy and photothermal efficacy. Andreou et al. demonstrated that SERS nanoparticles can accurately identify liver tumors and microscopic lesions in the liver and spleen with high contrast and high-resolution [45].

Tip-enhanced Raman spectroscopy (TERS) combined with a high-resolution spatial scanning probe microscopy can achieve single-molecular level surface chemical characterization with nanometer level spatial imaging resolution. A sharp metallic TERS tip is the critical component positioned at the excitation laser focus point to generate the EM field at the tip-apex for Raman signal enhancement (the working principle is the same as the SERS) [28]. TERS is widely used to investigate the chemical composition and the molecular dynamics of biological samples in aqueous mediums, such as pathogens, lipids, nucleic acids, proteins, and peptides [46,47].

Coherent anti-Stokes Raman spectroscopy (CARS) is a nonlinear optical spectroscopy technique which employs two excitation lasers with different frequencies to irradiate the sample simultaneously, a pump laser with frequency $\omega_{p}$, and a Stoke laser with frequency $\omega_{s}<\omega_{p}$. The frequency difference between two incident laser beams is equal to the chemical bond vibrational frequency of the sample. The interaction between the laser pulses and the molecular in the sample leads the four simultaneous vibrational coherences, a coherent anti-Stokes Raman scattering with frequency $(\omega_{p}-\omega_{s})+\omega_{p}$, a coherent Stokes Raman scattering with frequency $\omega_{s}$, a Raman gain with frequency $\omega_{s}$, and a Raman loss with frequency $\omega_{p}$. CARS has been widely used for the testing of lipids and proteins in cells and the characterization of the myelin sheath in nervous systems [28].

Stimulated Raman spectroscopy (SRS), also known as femtosecond stimulated Raman spectroscopy (FSRS), is a nonlinear optical technique with ultrafast sensing speed and high spectral resolution [48]. SRS uses three-pulse lasers to stimulate the sample, and the Raman spectrum is measured at different time intervals using a series of a narrowband Raman pump pulses and a broadband probe pulse. The generation of macroscopic polarization by the broadband probe pulse leads to free induction decay. SRS is a powerful tool for reaction dynamics due to its ultrafast sensing speed, and it has been applied to investigate the charge transfer and transport processes in solid-state materials [48,49,50].

Raman spectroscopy has many potential applications in regenerative medicine, including the analysis of cell differentiation, tissue engineering, and drug delivery. By providing a non-invasive and non-destructive method for analyzing molecular composition, Raman spectroscopy can help researchers better understand the underlying mechanisms of tissue regeneration and develop new therapies for human patients. In the future, Raman spectroscopy may be used to monitor the effects of therapy non-invasively and to discriminate between samples based on their molecular composition. This could greatly benefit the field of regenerative medicine by enabling novel therapeutic and diagnostic techniques to be implemented in humans [15].

### 2.2. Infrared Spectroscopy

**Figure 3:**
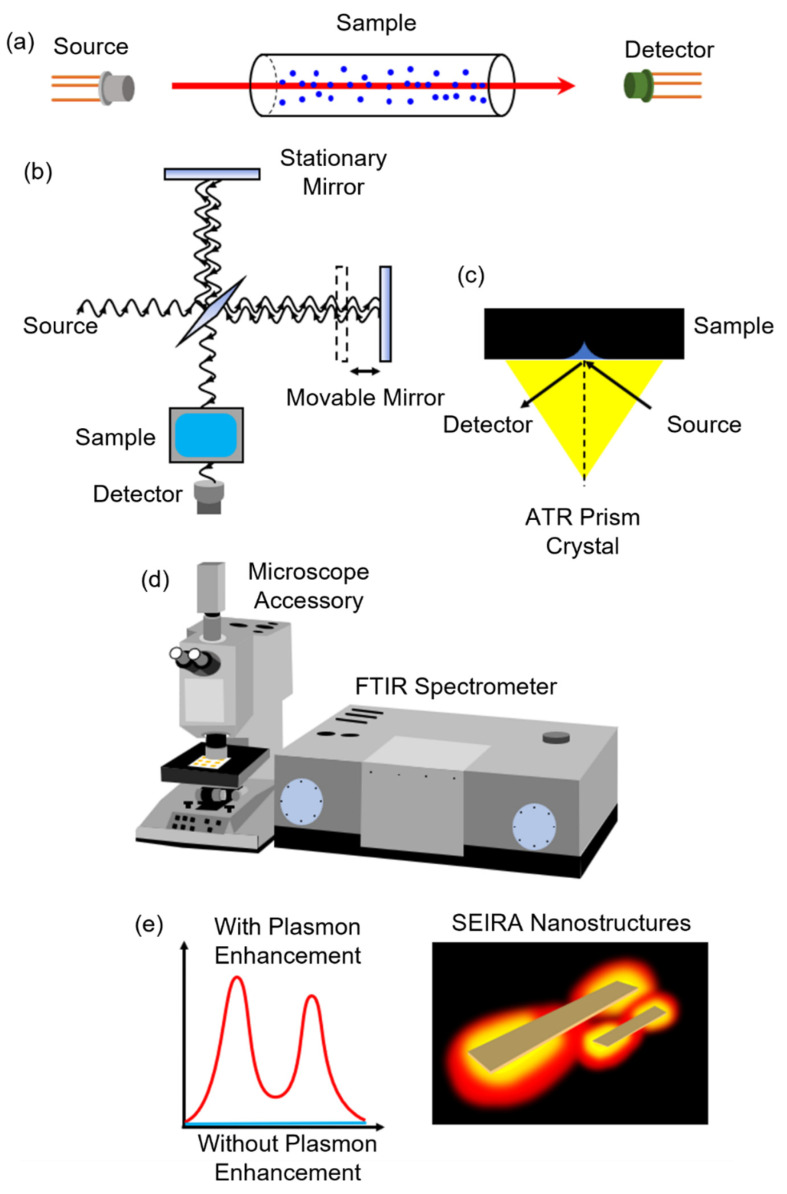
Illustrations of infrared spectroscopy techniques for (**a**) LAS, (**b**) FTIR spectroscopy, (**c**) ATR-FTIR spectroscopy, (**d**) infrared microspectroscopy, (**e**) SEIRA spectroscopy.

Infrared spectroscopy is a technique that uses infrared light to study the vibrational and rotational motions of molecules. There are several different types of infrared spectroscopy, each of which uses a different technique to measure the infrared absorption of a sample. Some of the most common types of infrared spectroscopy include [51]:

Laser absorption spectroscopy (LAS), as shown in Figure 3a: This technique uses a laser source on a sample and measures the reflected or transmitted light directly [52]. Usually, the sample is gaseous and is contained in a cell with two reflective mirrors to improve the interaction length. The laser is tuned to a specific wavelength that matches the vibrational frequency of the sample molecules, causing them to absorb some of the incident light energy. The intensity of light absorbed by the sample can be utilized to identify the type and measure the concentration of the sample.

Fourier transform infrared spectroscopy (FTIR), as shown in Figure 3b: The use of a Michelson interferometer to measure the infrared absorption of a sample allows for the determination of the sample’s chemical composition [53]. A broadband infrared light source is directed to a beam splitter, which ideally splits the light into equal parts. Half of the light reflects towards a stationary mirror, while the other half passes through a movable mirror. The two beams are then reflected back to the beam splitter, and the recombined light is focused onto the sample. The light is then refocused onto the detector after leaving the sample compartment. An interferogram is obtained by varying the difference in the optical path length between the two arms of the interferometer and recording the signal from the detector. A Fourier transform algorithm turns the interferogram data (light absorption for each mirror position) into the infrared absorption spectrum (light absorption for each wavelength) of the sample. The FTIR can provide quantitative information about the chemical composition of a wide range of samples, including solids, liquids, and gases as a function of light frequency with high resolution and sensitivity.

Attenuated total reflectance (ATR) FTIR spectroscopy, as shown in Figure 3c: This sampling technique works by shining infrared light through a crystal which is internally reflected at least once at the crystal-sample interface by total internal reflection [54]. During this reflection, a portion of the light enters the sample, where it can be absorbed, creating an evanescent wave. The penetration depth of the evanescent wave into the sample is determined by the difference in refractive indices between the sample and the ATR crystal. The evanescent wave propagates into the sample and interacts with it, allowing for the identification and quantification of the sample’s chemical components. ATR-FTIR spectroscopy is a non-destructive and easy-to-use technique suitable for analyzing solid, liquid, and gel samples. The crystal could be put on the fiber tip. The fiber probe-based FTIR sensing technique is suitable for remote sensing in hard-to-access areas and for real-time monitoring of the dynamic processes of biomarkers.

Infrared microspectroscopy, as shown in Figure 3d: This technique uses a microscope to focus infrared light onto a small sample area and measure the resulting absorption [55]. It allows for the analysis of microscale samples, such as single cells, tissue sections, or small particles, which may be too small or heterogeneous for conventional infrared spectroscopy. Infrared microspectroscopy can provide spatially resolved information about the composition, structure, and function of biological or material samples, which can be correlated with imaging or other analytical techniques. 

SEIRA spectroscopy, as shown in Figure 3e: This technique is based on the phenomenon of surface enhancement, where the absorption of infrared light by the sample is increased due to the interaction of the sample with the nanostructures typically on a metal or metal oxide surface [56]. SEIRA spectroscopy is particularly useful for analyzing samples that have low intrinsic absorbance in the infrared region, such as thin films or monolayers. Since the SEIRA structures are microscale or nanoscale, it relies on an infrared microspectroscopy technique to detect the reflected or transmitted signals. The resulting spectrum can be used to identify the chemical bonds and structures of the molecules at the surface of the sample.

The accuracy and reliability of infrared spectroscopy depends on the quality and preparation of the sample [53,54,56]. For instance, in FTIR spectroscopy, the sample must be free of contaminants, as even small impurities or variations in the sample can lead to significant errors in the spectra. In ATR spectroscopy, the contact between the crystal and the sample must be tight and stable, as any air gaps or movements can affect the intensity and shape of the spectra. In SEIRA spectroscopy, the size, shape, and density of the nanostructures on the surface can affect the enhancement and reproducibility of the spectra. In addition, interpreting the spectra obtained from infrared spectroscopy requires knowledge of and experience in chemistry and spectroscopy [57]. The spectra can contain a wealth of information about the functional groups, vibrations, and interactions of the molecules in the sample, but they can also be complex and overlapping. Various software programs and databases, such as IR spectral libraries and chemometric tools, can assist in spectral interpretation and analysis.

Overall, infrared spectroscopy is particularly useful for studying the vibrational and rotational motions of molecules that contain polar covalent bonds, such as C-O, C-N, and N-H bonds [53,57]. These bonds have a dipole moment, which allows them to interact with the incident infrared radiation. In addition, IR spectroscopy is also useful for analyzing molecules with functional groups, such as alcohols, amines, and carboxylic acids, which have characteristic absorption bands in the infrared spectrum. Raman spectroscopy, on the other hand, is particularly useful for analyzing molecules that have polarizability and undergo changes in polarizability upon excitation, such as C-C, C-H, and N-H bonds [58,59]. Raman spectroscopy is also useful for studying the orientation and conformation of molecules in a sample. The technique is particularly sensitive to changes in bond length and bond angle, which can be used to study molecular vibrations and rotations. While both infrared and Raman spectroscopy can provide information about the chemical composition and structure of a sample, they are each particularly useful for studying different types of molecules and chemical bonds [60,61]. For example, infrared spectroscopy is limited by the presence of water in the sample, but Raman spectroscopy is not. Water has a broad absorption band centered around 3450 cm^−1^, which corresponds to the stretching vibration of the O-H bond. Water also shows a relatively strong absorption band around 1600 cm^−1^, which corresponds to the bending vibration of the O-H bond. This can lead to significant interference and distortion in the spectra, making it difficult to analyze the sample. Several methods have been proposed to overcome this challenge, such as drying the sample or exchanging the water with deuterated water, which has a weaker absorption in the mid-infrared range. However, this may not always be feasible or practical for certain samples. Recent developments have demonstrated the potential of using multi-modal vibrational spectroscopy techniques to provide complementary and more comprehensive information about samples [62].

## 3. Cancer Diagnosis

Based on our literature survey, Raman spectroscopy is more commonly used for cancer diagnosis than infrared spectroscopy. Recent research has shown that Raman spectroscopy can be a promising tool for cancer diagnosis [18,63,64,65,66,67]. It can provide real-time information on cancerous tissues, distinguish between normal and cancerous tissues, and assess the efficacy of anticancer drugs [67,68]. Nevertheless, both Raman spectroscopy and infrared spectroscopy techniques can reduce the need for unnecessary biopsies, save medical resources, and improve the patient experience. They have been demonstrated for the detection of different types of cancer.

### 3.1. Prostate Cancer

Prostate biopsy procedures and the intra-operative assessment of tumor resection margins can be guided using Raman spectroscopy. For in vitro studies, the technique has been shown to differentiate between benign samples and prostate cancer with an overall accuracy of 86% [20]. Gaba et al. conducted a study that utilized Raman spectroscopy to distinguish between prostate and non-prostate tissue with an accuracy rate of 82% sensitivity and 83% specificity. The study also demonstrated that the technique can differentiate between benign and malignant prostatic tissue with an accuracy rate of 87% sensitivity and 86% specificity [69,70]. 

Haroon et al. explored the potential of SERS for prostate cancer diagnosis through various methods, including a SERS-based immunoassay and electrochemical-SERS (EC-SERS) method [71]. The main characteristic peaks for prostate cancer in SERS are shown in Table 1. The study by Mistro et al. found that SERS can be used to distinguish between the urine samples of healthy donors and those collected from patients with prostate cancer with a sensitivity of 100%, a specificity of 89%, and an overall diagnostic accuracy of 95% [72]. SERS can be combined with chemometric techniques such as principal component analysis (PCA) and linear discriminant analysis (LDA) to improve the accuracy of diagnosis [72]. 

P. Crow et al. developed a fiberoptic Raman system to differentiate between benign and malignant bladder and prostate pathologic findings in vitro [73]. The bladder and prostate algorithms could distinguish between malignant and benign samples with an accuracy of 84% and 86%, respectively. 

Kast et al. conducted a review of emerging techniques that can be used for the diagnosis of prostate cancer. They suggested that novel techniques such as shifted excitation Raman difference spectroscopy and TERS can improve the Raman signature by reducing noise and enhancing the signal. Moreover, non-invasive and non-destructive techniques such as atomic force microscopy and near-field scanning optical microscopy can be employed for imaging, while Raman spectroscopy can be used for molecular and chemical analysis [74]. 

According to these studies, Raman spectroscopy can provide accurate and non-invasive diagnostic information, as well as the ability to identify the biomarkers associated with prostate cancer. The scope of its applications in the field of prostate cancer is vast and includes tissue diagnosis, margin assessment, therapeutic development, and basic scientific research.

Extracellular vesicles are small membrane-bound structures secreted by cells and that are associated with malignancy. They can contribute to resistance to cancer treatment and angiogenesis. Urinary extracellular vesicles (UEVs) have the potential to be a non-invasive biomarker for detecting prostate cancer, as they may contain a distinctive set of biomolecules that can be utilized as a signature profile. UEVs can improve the specificity and sensitivity of prostate cancer detection compared to invasive methods such as biopsies. Yap et al. aimed to investigate the potential of UEVs as a non-invasive biomarker for prostate cancer detection using ATR-FTIR spectroscopy [75]. The spectra were analyzed using PCA and LDA. The study analyzed urine samples from 12 prostate cancer patients and 12 healthy individuals. It was observed that the spectral peaks in the urine exosomes of prostate cancer patients differed from those of healthy individuals at specific wavelengths, such as the amide I peak (1640 cm^−1^), RNA ribose peak (1120 cm^−1^), and C-N stretch peak (967 cm^−1^), among others. The resulting diagnostic classifier for prostate cancer achieved a sensitivity of 83.33% and a specificity of 60%. The study sheds light on the potential of UEVs as a non-invasive biomarker for prostate cancer detection using IR spectroscopy, which may increase the specificity and sensitivity of prostate cancer detection compared to traditional methods that require invasive procedures such as biopsies. 

Krafft et al. aimed to identify a distinct spectral pattern of extracellular vesicles derived from the serum and plasma for cancer screening [76]. To achieve this, they performed a comprehensive comparative analysis of extracellular vesicles using both infrared and Raman spectroscopy. This analysis was conducted on extracellular vesicles from both cancerous and non-cancerous sources, as well as on samples from patients being screened for cancer. The researchers identified several spectral features that were significantly different between the two groups, especially peaks associated with proteins.

### 3.2. Skin Cancer

Cancerous skin tissues have different molecular compositions compared to normal skin tissues. A study found that regenerative skin has a significant increase in cellular density, nucleic acid content, neutral lipid density, Collagen III, and glycosaminoglycans compared with reparative skin [70]. Lipids are the main component of cellular membranes and are highly diverse in structure [77]. Nucleic acids such as messenger RNAs (mRNAs), antisense oligonucleotides (ASOs), and short interfering RNAs (siRNAs) hold great promise for treating previously ‘undruggable’ diseases [78]. Raman spectroscopy can be used for the in vivo characterization of skin lesions in real-time due to its non-intrusive nature, the absence of sample preparation, and its high chemical specificity [79]. Raman spectroscopy has been used in several studies to diagnose different types of skin cancer with high sensitivity and specificity [80]. Using Raman spectroscopy, it is possible to detect the biochemical differences between basal cell carcinoma (BCC) and normal skin structures with high sensitivity, which can be used for the development of a diagnostic model. 

Feng et al. introduced a biophysical model for human skin cancer that allows for the inference of the skin’s biochemical composition based on its Raman spectrum [81]. They also demonstrated the use of superpixel acquisition for the rapid discrimination of BCC tumors from normal skin. The findings indicate that there is a strong ability to differentiate between tumor and normal skin using variations in the nucleus, collagen, keratin, and ceramide. Moreover, the faster processing speed of the superpixel method is directly proportional to the ratio of the superpixel area to the laser spot size. With this approach, it is estimated that scanning a tissue sample of 1 × 1 cm would take 2.7 h [82].

A study carried out by Lieber et al. showcased the effectiveness of a portable Raman probe in distinguishing between benign lesions and malignant/premalignant lesions with high sensitivity rates ranging from 95% to 99% [83]. Another study by Nijssen et al. used a two-step classification model based on Raman spectra to diagnose BCC with 100% sensitivity and 93% specificity [84]. 

A novel approach that combines auto-fluorescence and Raman spectroscopy has been developed to accelerate the diagnostic process while maintaining high accuracy [85]. AF-Raman was employed by Kong et al. to detect BCC in skin resections obtained during Mohs surgery, and they provided illustrative instances [20]. This approach accurately assessed skin resections with only 500–1500 Raman spectra. Combining fluorescence and Raman spectroscopy presents a promising non-invasive technique for detecting malignant skin cancer in humans. Fluorescence analysis enables quick scanning of extensive tissue areas, while Raman spectrum analysis can be utilized to identify suspected cases of malignancy and determine the type of tumor. 

To identify skin tumors in vivo, a novel RS phase method has been suggested, which utilizes quadratic discriminant analysis for tumor type classification. The combined diagnostic method is shown to be highly efficient, with a sensitivity of 89% and a specificity of 87% in identifying malignant melanoma [86]. Singurdsson et al. developed a skin lesion classification method using in vitro Raman spectroscopy and a nonlinear neural network classifier. Their approach is fully automated and probabilistic, including feature extraction and a feedforward neural network that is fully adaptive. The study results showed a correct classification rate of 80.5% for malignant melanoma and 95.8% for basal cell carcinoma. The overall classification rate of skin lesions was 94.8% [87]. 

Khristoforova et al. presented a portable Raman spectroscopy system for skin tumor optical screening with 100% accuracy in the differentiation of benign and malignant skin tumors through a combined analysis of Raman and AF signals [88,89].

**Figure 4:**
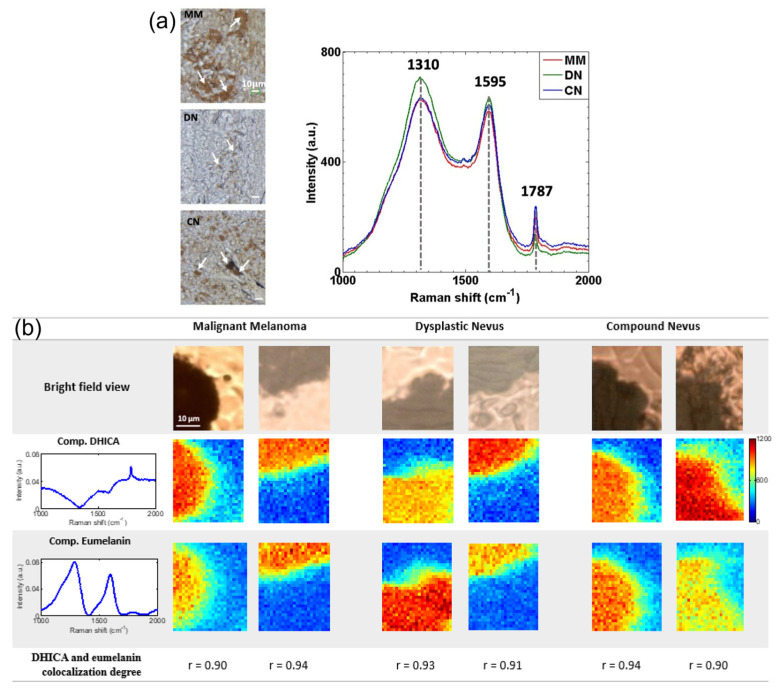
“(**a**) Raman spectra acquisition in the dark−pigmented regions of tumors: malignant melanoma (MM), dysplastic nevus (DN) and compound nevus (CN) lesions.” (**b**) Raman spectral maps of skin lesions. (Adapted with permission from Ruiz et al.) [91].

Collagen and triolein have proven to be the most relevant biomarkers for diagnosing melanoma and non-melanoma skin cancers. Raman spectroscopy could be used to discover the biophysical basis for accurate diagnosis, potentially reducing the need for excisional biopsies [90]. Ruiz et al. devised a novel methodology to non-invasively and rapidly quantify and localize biomarkers that classify dysplastic lesions with a sensitivity of 94.1% and a specificity of 100%. The Raman spectral maps are shown in Figure 4 [91].

Tamosiunas et al. employed ex vivo Raman spectroscopy on various tissue samples including soft tissue sarcomas, lipomas, skin, and mast cell tumors taken from cats and dogs. The study combined OCT and Raman data, resulting in better differentiation between the samples. The method achieved high sensitivities for skin, lipomas, and malignant tumors, with values of 0.968, 1, and 0.939, respectively. The specificities were also high, with values of 0.956, 1, and 0.977 for skin, lipomas, and malignant tumors, respectively [92]. 

Liao et al. have developed a new technique for Raman spectroscopic imaging that enables the real-time analysis of cellular processes and states without the need for labeling. This approach uses parallel detection, which allows for the mapping of lipid droplets in individual live cells, the examination of retinoid metabolism, differentiation between fat droplets and protein-rich organelles, and the monitoring of drug diffusion in vivo [93]. 

Bratchenko et al. explored the potential of a cost-effective and portable spectroscopy setup for in vivo skin cancer diagnosis. They achieved a biopsy ratio of 3.95:1 with 90% sensitivity for Raman classification, and a biopsy ratio of 3.33:1 with 90% sensitivity for autofluorescence classification [89]. Two methods for classifying skin cancer based on Raman spectra analysis were compared by the researchers: convolutional neural networks and projection on latent structures with discriminant analysis [94]. The success of classifying skin tumors with high accuracy using convolutional neural network analysis indicates the potential for widespread adoption of Raman setups in clinical settings.

Current studies are investigating the potential of Raman spectroscopy for in vitro cancer diagnosis, with recent research focused on the development of portable systems for the real-time measurement of skin lesions and the analysis of serum for gastric cancer [42,81,95]. The accurate correlation between Raman spectra and enzymatic test results, as well as the differentiation between healthy and cancerous tissues, heavily relies on the use of multivariate data analysis techniques, including partial least squares regression and partial least square discriminant analysis.

Farries et al. reported a prototype using MIR spectral imaging for the quick assessment of cells for cytological diagnosis [96]. The prototype, known as the Minerva, was created based on a fiber optic supercontinuum source with large spectral brightness, coupled with an acousto-optic tunable filter that collects data across a wavelength range selected for higher sensitivity for a specific skin disease. The Minerva was tested with colon cells across a wavenumber range of 2700 to 3500 cm^−1^, and proved to be advantageous over the state-of-the-art FTIR system since it could capture spectral data about 500 times faster. The increased speed in data collection can potentially allow for real-time screening in vivo, removing the need for biopsy and the long wait time required for histopathological analysis. In addition, the Minerva could produce high-resolution images and create three-dimensional images with phase information.

Kyriakidou et al. used ATR-FTIR spectroscopy to analyze the spectral differences between healthy skin tissue and skin tissue affected by basal cell carcinoma, malignant melanoma, and nevus for the early clinical diagnosis of skin cancer [97]. Seven patients were studied and normal skin tissues adjacent to the affected regions were used as the control. The study found characteristic “marker bands” for proteins, lipids, and nucleic acids. For example, the intensity at approximately the 3062 cm^–1^ band was increased upon cancer development, indicating the predominance of the β-sheet protein structure, and two absorption wavenumbers at 841 cm^−1^ and 815 cm^−1^, which correspond to B- and Z-DNA forms, respectively.

### 3.3. Gastric and Colorectal Cancer

Despite being the preferred screening method for colorectal cancer (CRC), colonoscopy is not widely adopted due to its high cost and practical limitations, even though CRC is the third most prevalent cancer worldwide and is also a significant contributor to cancer-related deaths [98]. Raman spectroscopy has demonstrated high sensitivity and specificity in differentiating normal mucosa, adenocarcinomas, and adenomatous and metaplastic polyps, in vitro as well as in vivo. It has also been used to monitor tumor progression in mice by measuring molecular changes in the tumor. High-frequency Raman spectroscopy has discriminated between pathology subtypes in fresh ex vivo colon tissue samples with one second acquisition times. Raman spectroscopy could be used as a supplementary tool to histopathology, either for automated sample analysis or the digital staining of tissue sections to aid pathologists in their analysis [20]. Lin et al. presented findings on the capabilities of SERS to obtain blood serum biochemical information for the early detection of colorectal cancer [99]. SERS measurements were able to identify characteristic biomolecular changes associated with colorectal cancer, such as changes in protein and lipid concentrations. The major vibrational bands for the colorectal cancer serum samples are shown in Table 2.

**Figure 5:**
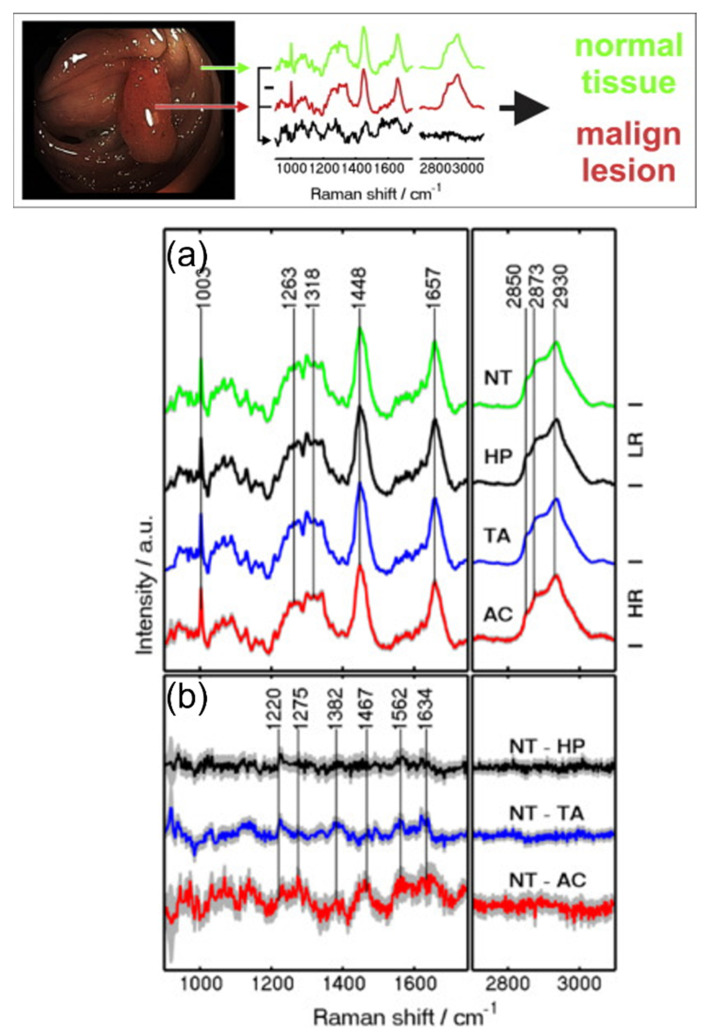
Raman spectra by fiber−optic measurements. (**a**) Mean spectra of normal tissue (NT), hyperplastic polyp (HP), tubular adenoma (TA) and adenocarcinoma (AC). (**b**) The difference spectra of NT with HP, TA and AC. (Adapted with permission from Petersen et al.) [101].

Ito et al. devised a rapid and straightforward process for producing silver nanoscale hexagonal columns on a phosphor bronze chip, which could be used for SERS measurements. Their findings indicated that the SERS spectra’s peak heights were markedly lower in patients with benign diseases than in those with CRC [100]. Petersen et al. used a fiber-optic Raman probe to diagnose CRC. The samples included normal tissues, hyperplastic polyps, tubular adenomas, and adenocarcinoma tissues. The Raman spectra of low-risk (LR) and high-risk (HR) lesions of different tissues are shown in Figure 5 [101]. Wang et al. devised a label-free technique for detecting serum proteins using surface-enhanced Raman spectroscopy (SERS). Their results showed alterations in the secondary structures and amino acid contents of serum proteins during cancer progression [38].

Salman et al. showed that FTIR spectroscopy could differentiate between control, local and distant recurrence crypts in CRC relapse [102]. CRC relapse is common, with more than half of CRC patients experiencing it. A total of 128 crypts from eight CRC patients were used in this study, where 21 were classified as no recurrence, 53 were classified as local recurrence, and 54 were classified as distant recurrence. These samples were formalin-fixed and paraffin-embedded (FFPE), and FTIR spectral data was collected from 600 to 4000 cm^−1^. The data sent for PCA and LDA were used in helping to differentiate between the classes of tissue samples. Employing the principal components recognized, a 92% accuracy rate in distinguishing between control, local recurrence, and distant recurrence crypts was attained. 

**Figure 6:**
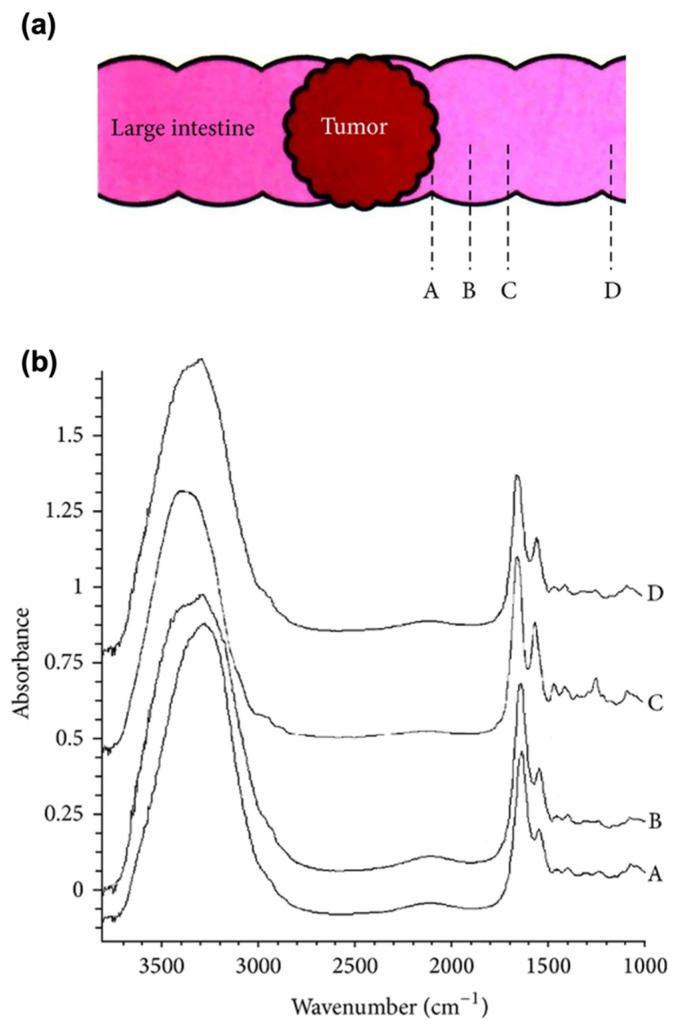
(**a**) The schematic of CRC specimens and the samples obtained from four parts (Part A: tumor sample, Part B: intestinal mucosa sample 1 cm away from the tumor, Part C: intestinal mucosa sample 2 cm away, and Part D: intestinal mucosa sample 5 cm away). (**b**) FTIR spectra at specific locations in (**a**). (Adapted with permission from Yao et al.) [103].

Yao et al. described a pilot study on the use of ATR-FTIR for evaluating surgical resection margins in CRC [103]. The samples were fresh tissue samples collected from 56 patients with CRC. It was found that the FTIR spectra of CRC and adjacent mucosa at 1 cm differed from those at 2 cm and 5 cm, as shown in Figure 6. The FTIR analysis revealed a reduction in lipids and a rise in protein and nucleic acid content in both the tumor and surrounding tissue within a 1 cm range. These findings suggest that FTIR spectroscopy has potential as a quick and effective means of assessing surgical resection margins in cases of colorectal cancer.

Nallala et al. studied the area of identifying gastrointestinal (GI) cancers using MIR spectral imaging [104]. It is known to be difficult to achieve inter and intra-observer agreement for diagnosis in the early stages of GI cancer. Fifty tissue samples were measured in this study, while 45 samples were analyzed: 16 were non-tumoral, 13 were tumoral, 7 were adenoma, and 9 were hyperplastic. FTIR spectral data was collected using an Agilent 620 FTIR microscope, which was coupled with an Agilent 670 FTIR spectrometer equipped with a Globar light source. The microscope had a liquid-nitrogen cooled focal plane array (FPA) detector for collecting the spectral data. The samples were measured using the conventional pixel resolution of 5.5 × 5.5 μm^2^, and then smaller regions were imaged using a higher resolution modality of pixel resolution 1.1 x 1.1 μm^2^. The collected data underwent multivariate analysis, including PCA-LDA, paired with leave-one-out-cross-validation (LOOCV) to build a classification model. Using the classification model with two groups: tumoral and non-tumoral, sensitivity and specificity values ranged from 81 to 86%, with increased performance from conventional to high-resolution images. The addition of the intermediate group, including adenoma and hyperplastic tissues, resulted in a drop in model performance. 

Sheng et al. used FTIR spectroscopy as a potential diagnostic tool for gastric cancer [105]. Serum samples were collected from gastric cancer patients (27 cases before surgery) and healthy individuals (19 cases), and FTIR spectroscopy was used to analyze their biochemical compositions. An additional 12 samples were included for blind tests. A test accuracy of 100% was achieved. The study also found that the H2959/H2931 ratio was the most efficient in distinguishing gastric cancer patients from healthy individuals. The RNA/DNA ratio was lower in gastric cancer patients than in healthy individuals. 

Kaznowska et al. proved that ATR-FTIR spectroscopy could provide valuable information on the biochemical composition of samples of colon tissues, potentially serving as a pathophysiological tool to monitor the effectiveness of chemotherapy treatment for cancer patients [106]. A total of 56 paraffin-embedded colon specimens were used in the study and were histologically analyzed and classified into several categories: healthy, cancerous, post-chemotherapy cancerous, and healthy surgical margin. FTIR measurements with a diamond ATR crystal were taken in the range of 400–4000 cm^−1^, but only data in the range of 900–3500 cm^−1^ were selected. The obtained data were then analyzed using PCA-LDA. A comparison between different tissue classes showed that the cancerous samples did not show peaks belonging to functional groups in nucleic acids (1085 cm^−1^, 1249 cm^−1^), proteins (1550 cm^−1^, 1648 cm^−1^), and water (3250 cm^−1^). Additional vibration at 1385 cm^−1^ was found to be present in cancerous, post-chemotherapy cancerous, and healthy surgical margins of CRC. The differences in the biochemical composition of the tissue types could potentially allow FTIR to become a tool in determining the margin of the tumor for clean and proper resection of the tumor.

### 3.4. Breast Cancer

Normal and cancerous breast tissue differs primarily in their lipid and protein contents, with normal breast tissue containing higher levels of lipids and cancerous tissue containing higher levels of proteins. Raman spectroscopy has demonstrated the potential in the non-invasive detection, grading, and classification of breast cancer according to research findings [107]. Raman spectroscopy technology provides label-free, real-time, and non-subjective chemical information that can differentiate between malignant and benign tissue. Raman probes enable the in vivo acquisition of Raman spectra with an accuracy rate of 93.3% for in vivo measurements. [20]. Raman biomarkers such as carotenoids, proteins, lipids, nucleic acids, collagen calcium compounds, and water can be utilized to identify the primary differences between normal and cancerous tissue [20,107]. Raman spectroscopy has been shown to have a higher sensitivity in detecting micro-calcifications when compared to mammography. This increased sensitivity reduces the likelihood of false negatives and miss-sampling. Furthermore, the SORS technique offers a non-invasive method for analyzing calcifications at various depths in both human and chicken breast tissue. 

**Figure 7:**
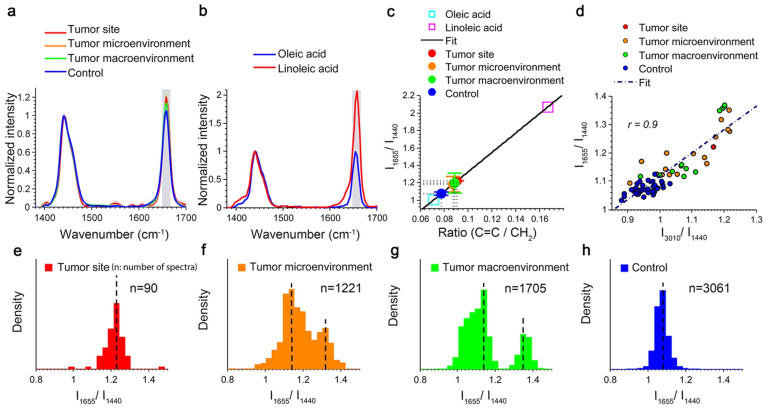
(**a**) Comparison of the average Raman spectra of fatty acids, (**b**) Raman spectra of two pure reference fatty acids (oleic acid and linoleic acid), (**c**) Quantitative analysis of fatty acids from different tissue sites, (**d**) Correlation plot of Raman intensity at 1665 cm^−1^ and 3010 cm^−1^ from fatty acids, (**e**–**h**) Density distributions of the degree of unsaturation of fatty acids from four groups. (Adapted with permission from You et al.) [108].

The study by You et al. employed Raman micro-spectroscopy to investigate the impact of fatty acids on cancer cell growth and metastasis. Their findings showed a significant change in the tumor, as there was a shift from monounsaturated fatty acids to PUFAs on a large scale, while only a minor subset of fatty acids underwent this transition in the tumor’s micro- and macro-environment, as shown in Figure 7 [108]. 

A multivariate partial least squares regression model was developed by Bilal et al. based on the Raman spectra of BRC-positive and healthy participants. The model’s R-square value was 0.987, indicating promising results in terms of accuracy, sensitivity, specificity, and the receiver operating characteristic curve [109].

Raman imaging of breast samples demonstrated excellent contrast, attributed to the existence of proteins, fatty acids, and carotenoids, with a more significant protein contribution observed in cancerous samples, while normal breast tissue contained higher levels of lipids, particularly oleic acid derivatives. An analysis of nodal sections using Raman mapping revealed the potential for a comprehensive assessment of the biochemical modifications linked to metastasis [110]. The use of Raman spectroscopy was applied to examine the proliferation status of rat cells, demonstrating that actively dividing cells have a greater concentration of nucleic acids and proteins. Furthermore, a Raman spectroscopy analysis of serum samples obtained from breast cancer patients indicated changes in the intensity and peak positions of various bands, such as beta-carotene, proteins, polysaccharides, and phospholipids. By using LDA, discrimination between control and cancerous samples was achieved with a sensitivity of 92.2% and a specificity of 78% [110]. The amide region was identified as the main area of difference between normal and diseased breast tissue analyzed using FT-Raman spectroscopy, which is possibly attributable to the poor scattering caused by the irregular surface of the tissue.

SERS has also been utilized for the detection of oncogenes at low concentrations ranging from micro to picomolar levels. The application of nanoparticle SERS probes has enabled the identification and targeting of breast tumor components, facilitating the characterization and evaluation of tissue both prior to and post-therapy [65,110]. SERS has also been used to target HER2 on breast cancer cells and to identify other cancer markers such as PSA, BRCA1, EGFR, and others. The use of gold nanoparticles in serum research has also been explored, resulting in a 96% sensitivity and 87% specificity in detecting breast cancer [110]. 

Ozek et al. showcased their method for examining comprehensive alterations in cells as a result of miRNA expression using a model cell line system [111]. The researchers conducted a study using MCF7 cells to investigate the effects of miRNA-125b, which is known to be down-regulated in breast cancer. Specifically, they compared cells transfected with miRNA-125b (MCF7-125b) to cells transfected with an empty vector (MCF7-EV). The study utilized ATR-FTIR and spin-labeling electron spin resonance spectroscopy to examine the global structural changes of the cells over time. The results indicated that MCF7-125b cells exhibited lower levels of RNA, protein, lipid, and glycogen content, as well as lower membrane fluidity and proliferation compared to MCF7-EV cells. Using these changes as features, cluster analysis was used to differentiate between MCF7-125b and MCF7-EV cells, demonstrating a promising approach for understanding the effects of miRNA on cells. This methodology could potentially be applied to diagnose deregulated miRNA expression in patient samples in the future.

Tomas et al. demonstrated the effectiveness of neural networks trained using the ATR-FTIR spectral data of breast tumors [112]. FFPE breast tissue blocks were obtained from 166 patients, and 78 tissue blocks were histopathologically classified as benign, while 88 were classified as malignant. A platinum ATR single reflection diamond sampling module was used, and data from the wavenumber range of 600–4000 cm^−1^ was collected. The data underwent several pre-processing steps via removing background noise, baseline correction, and Z-score normalization before being processed by ML. The classification models included PCA-LDA, SVM, decision tree, random forest, naive Bayes, logistic regression, deep learning models, and feed-forward neural networks (FNN) of different hyperparameters. The best model was the FNN, with two fully connected layers with 96.06% accuracy. The FNN method was used to determine that the significant regions belonged to C–OH functional groups in carbohydrates. 

Yang et al. conducted a study with the aim of using the FTIR spectroscopy of serum along with machine learning algorithms such as SVM, KNN, and extreme learning machines (ELM) to classify breast cancer patients at different stages and non-cancer control subjects [113]. They used serum samples from 120 breast cancer patients at different stages and 60 non-cancer control subjects for the analysis. The study revealed that the technique could quickly and effectively distinguish between breast cancer patients at different stages and non-cancer control subjects. The accuracy rate for distinguishing between stage 1 breast cancer patients and non-cancer control subjects was found to be 96.7%, while the accuracy rate for distinguishing between stage 2–4 breast cancer patients and non-cancer control subjects was 100%. The study offers a promising approach to breast cancer screening that is both efficient and cost-effective, potentially allowing for the earlier detection and treatment of breast cancer in a larger population. Additionally, this approach may be applicable to other types of cancer screening through serum FTIR spectroscopy in the future. 

Liu et al. investigated the feasibility of using FTIR spectroscopy combined with SVM as a screening tool to identify invasive ductal carcinoma (IDC) in breast cancer [114]. The samples were serum samples from healthy patients, IDC patients, and non-IDC patients. It analyzed a total of 180 serum samples, including 60 healthy controls, 60 IDC patients, and 60 non-IDC patients. Specific differences in the infrared spectra of the three groups of serum were observed due to differences in the contents of specific substances, which led to changes in peak intensity and spectral shape. The results showed that the SVM algorithm had a high accuracy rate of 92.5% in identifying IDC. The sensitivity and specificity were 91.7% and 93.3%, respectively. The study presents a promising approach to detecting breast cancer using advanced technology that is rapid and noninvasive. 

Depciuch et al. proposed a physics-based computational model for analyzing FTIR spectra to assess the effectiveness of chemotherapy in treating breast cancer [115]. The study involved examining breast tissue samples from 33 females diagnosed with triple-negative breast cancer who underwent pre-operative chemotherapy. Two patients showed a partial response after four chemotherapy cycles. The team analyzed deparaffinized breast tissue specimens using ATR-FTIR spectroscopy, including pre-chemotherapy, post-operative, and post-chemotherapy specimens. The results revealed significant differences in the FTIR spectra between healthy and cancerous breast tissue, as well as between pre- and post-chemotherapy breast tissue. Cancerous tissue exhibited three unique peaks at 1051 cm^−1^ (vibration of glycogen-derived C-O bonds), 1417 cm^−1^ (protein-derived COO bonds), and 1645 cm^−1^ (the stretching of C-O and C-N bonds found in primary amide structures) that were absent in healthy tissue. On the other hand, healthy breast tissue showed peaks at 1411 and 1654 cm^−1^ wavenumbers. The results illustrate how computational models based on FTIR spectroscopy have the potential to monitor the effectiveness of chemotherapy treatment in breast cancer patients in a non-invasive manner. This could lead to improved diagnostic tools for assessing treatment efficacy during the course of therapy.

Multimodality sensing combining both technologies was also reported. For example, Depciuch et al. compared the differences between the measurements obtained from Raman and FTIR spectroscopy for healthy and cancerous breast tissues for paraffin-embedded and deparaffinized samples [116]. Tissue samples are often prepared by embedding the tissue with paraffin, which can result in background signals. A total of 16 samples of breast tissues were used for this study, including eight paraffinized and eight deparaffinized samples. This study compared the differences between paraffined and deparaffinized tissue samples by two spectroscopy modes: FTIR and Raman in the range of 500–3000 cm^−1^. Paraffin was measured as the background for paraffined samples, while the air was used as the background for deparaffinized samples. Through performing Gaussian and Lorentz analyses, the percentage of alpha-helices and beta-harmonica was determined, and the values for the paraffined and deparaffinized samples were similar, while the differences were statistically insignificant. Hence, sample preparation had no impact on the mid-infrared (MIR) range, and only had a minor effect on the intensity of the Raman peak. Deparaffinized tissues, however, provide higher intensities using the Raman spectroscopy method, which could be beneficial for extracting more useful information in the spectrum data. 

Another example was by Brozek-Pluska et al., who proved that the sample preparation method of tissue samples affects the spectrum measured [117]. This study analyzed the impact of sample preparation on the spectrum presented from two spectroscopy modes: infrared and Raman in the wavenumber range of 2800–3000 cm^−1^. A total of 18 breast tissue samples were used, with six fresh samples, six paraffin-embedded samples, and six deparaffinized samples from three patients. For the infrared mode, spectral data was captured using the Nicolet Avatar 330 FTIR Spectrometer. Gauss functions were implemented on the spectral data to find the expected characteristic frequencies of different sample types. Through this analysis, variations in the spectrums were observed between tissue samples that underwent different preparation methods. Paraffin-embedded samples were dominated by paraffin peaks, hence disturbing the biochemical composition of tissues. Deparaffinization of the samples would also affect the intensities of the different peaks in both IR spectra, reducing lipid hydrocarbon peaks, where the noncancerous fresh tissue is dominated by the peaks at 2854 cm^−1^ and 3009 cm^−1^ belonging to the unsaturated bonds of lipids; however, in the deparaffinized tissues, the intensity of the spectrum was much lower than expected. These methods of preparation hence affect the output spectrum presented, disturbing the spectra in such a manner that the biochemical composition of the tissues cannot be accurately identified.

In summary, vibrational spectroscopy techniques such as Raman spectroscopy and Fourier-transform infrared spectroscopy have potential advantages as tools for the clinical diagnosis of breast cancer [118]. These techniques can detect subtle biochemical changes relating to pathology and can be used in tissue diagnosis [118]. Raman spectroscopy can be used to analyze both ex-vivo tissue and liquid biopsy samples [119]. The combination of vibrational spectroscopy with AI creates a pathway with significant potential for predicting various stages of different disease processes, specifically in cancer diagnosis, staging, and treatment design [120].

### 3.5. Oral Cancer

The oral cavity is composed of various components such as the lips, the mucosal lining, the buccal cavity, the upper and lower alveolar ridges, gingiva, the hard palate, the retromolar trigone, the floor of the mouth, and the front part of the tongue [109]. In their work, Ibrahim et al. explored the possibility of using Raman spectroscopy as a diagnostic tool for oral cancer [121]. There are three majority biomarkers for the diagnosis of oral cancer that can be used [122]:

Deoxyribose: This is a component of DNA that is different in cancerous and healthy tissues.

Collagen: This protein provides tissue structure and is known to be altered in cancerous tissues.

Lipids: Lipids are a class of biomolecules known to be altered in cancerous tissues and that can be used as biomarkers for cancer diagnosis.

The detection of oral cancer, particularly in various sub-sites including the tongue, buccal mucosa, and gingiva, was examined by Jeng et al. using Raman spectroscopy. The most effective classifier model was found to be the PCA-QDA, which yielded an accuracy rate of 87.5%, with a sensitivity of 90.90% and a specificity of 83.33% [123]. The primary biomolecular markers for detecting oral cancer were found to be variations in protein, amino acids, and beta-carotene. Serum Raman spectroscopy is one approach in oral cancer screening. Several studies have demonstrated the possibility of detecting premalignant and cancer-specific indications with high levels of sensitivity and specificity. These rates have been reported to be as high as 64% and 80%, respectively, which are comparable to established screening technologies [124].

Zlotogorski-Hurvitz et al. investigated the potential of the ATR-FTIR spectra of salivary exosomes in the diagnosis of oral cancer [125]. The study involved collecting whole saliva samples from 21 cancer patients and 13 healthy individuals, isolating exosomes and measuring their IR absorbance spectra. Machine learning techniques were employed to develop discrimination models for the absorbance data, including the PCA–LDA and SVM classification. The results showed that the IR spectra of oral cancer exosomes were consistently different from those of healthy individuals, indicating specific IR spectral signatures for cancer salivary exosomes. The relative intensity ratios of I_1033/1072_, I_2924/2854,_ I_1404/2924_ could be used for classification with statistical significance. These bands are associated with fatty acids and proteins. The PCA-LDA discrimination model was able to classify the samples accurately, with a sensitivity of 100%, a specificity of 89%, and an overall accuracy of 95%. The SVM exhibited a training accuracy of 100% and a cross-validation accuracy of 89%. The study concludes that this non-invasive method should be further investigated for the diagnosis of oral cancer at its very early stages, or in oral lesions with the potential for malignant transformation.

### 3.6. Lung Cancer

**Figure 8:**
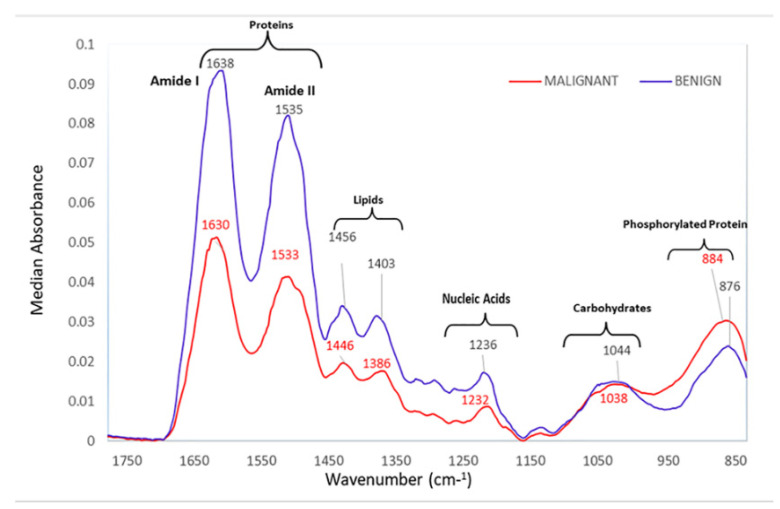
A representative spectrum of malignant and benign samples. (Adapted with permission from R. Bangaoil, et al.) [126].

Bangaoil et al. proved that ATR-FTIR was promising as an alternative to diagnosing lung cancer [126]. There were two primary lung cancer tumor categories: NSCLC and small cell lung carcinoma (SCLC). Both tumors were classified as malignant, and the non-cancerous samples were classified as benign. A total of 97 samples were used in this study, with 44 malignant samples and 53 benign samples. The tissue samples were obtained from a lung tissue block, where samples were cut from the outer sections and stained with hematoxylin and eosin. Samples cut from the inner section were deparaffinized with xylene. The spectral profiles of samples were measured in the range of 850–1800 cm^−1^ using a platinum single-reflection diamond sampling module. The spectra were then processed using PCA and hierarchical cluster analysis to differentiate between the tissue types. Figure 8 shows the five distinct spectral profiles that were identified, representing proteins, lipids, nucleic acids, carbohydrates, and phosphorylated proteins. Using the significant wavenumbers identified, a linear discriminant analysis (LDA) was conducted to develop a classification model for differentiation between malignant and benign samples. The model was tested to be 97.73% sensitive, 92.45% specific, 94.85% accurate, and 91.49% correct with regard to its positive predictions, and 98% correct with regard to negative predictions, with strong agreement observed with histopathologic classification.

Yang et al. proved that ATR-FTIR spectroscopy could be combined with chemometrics for a straightforward screening and diagnosis of lung cancer [127]. Samples were the blood serums of lung cancer patients and healthy patients, which were dried using a vacuum oven. In this study, 92 serum samples were collected from individuals with lung cancer, while 155 samples were obtained from healthy subjects. A deuterated triglycine sulfate (DTGS) detector was utilized to take ATR-FTIR measurements, wherein the pressure tip was used to press the dried serum sample. Raw data were pre-processed before being sent for a chemometrics analysis, including principal component regression (PCR) and partial least squares discriminant analysis (PLS-DA). The most prominent difference was identified in the first derivative of the spectral data, from 1000 to 1250 cm^−1^ and belonging to nucleic acids, in which the PLS-DA models were 80% sensitive, 91.89% specific, and 87.10% accurate. With the high performance indicated by the metrics and the low root mean square error (RMSE), ATR-FTIR spectroscopy with chemometrics is a promising and straightforward method for screening and diagnosis. 

Lugtu et al. investigated the potential of artificial neural networks in the discrimination of lung cancer based on ATR-FTIR spectroscopy [128]. In the case of lung cancer detection, this technique can identify specific molecular changes associated with cancerous cells. The advantages of using artificial neural networks were to improve the accuracy and efficiency of lung cancer diagnosis, especially in cases where pathologists may have discordant readings or uncertainties. This approach has been tested in clinical trials using lung tissue specimens on glass slides obtained from patients undergoing surgery for suspected lung cancer. A total of 70 samples were collected, including 39 malignant and 31 benign samples. The results showed that the artificial neural network models had high accuracy rates in discriminating between benign and malignant lung tissue samples based on infrared spectroscopy data. The best-performing model achieved an accuracy rate of 94.3%, a sensitivity of 94.9%, a specificity of 93.5%, a positive predictive value of 92.7%, and a negative predictive value of 95.6%. 

Kaznowska et al. proposed the use of ATR-FTIR spectroscopy combined with PCA-LDA and a physics-based computational model to classify lung cancers and determine their degree of malignancy [129]. The study used lung cancer tissue slices, including adenocarcinoma and squamous cell carcinoma, as well as control tissue samples, mounted on CaF_2_ slides for FTIR spectroscopic analysis. The findings revealed a shift in the spectral pattern of adenocarcinoma tissue compared to control and lung cancer tissues. Notably, the adenocarcinoma tissue spectra lacked peaks for functional groups of glutamate or phospholipids. The study concluded that FTIR spectroscopy, coupled with PCA-LDA analysis and the physics model, is a sensitive tool for not only diagnosing tumor types but also for classifying their malignancy. 

Exhaled breath analysis is a promising non-invasive approach for diagnosing and monitoring airway diseases. Mastrigt et al. developed a broadband quantum cascade laser (QCL) spectroscopy technique to detect volatile organic compounds (VOC) in exhaled breath samples [130]. They utilized a QCL and a multipass cell to evaluate the repeatability of the measurements for exhaled breath VOC profiling and to analyze its usability to differentiate 35 healthy subjects, 39 asthmatic patients, and 15 children with cystic fibrosis (CF). The authors showed that it was possible to differentiate classes of children with CF by analyzing the spectral profiles using PCA. A group of VOCs was identified between healthy children and children with asthma in wavenumber ranges of 1181.80–1182.55 cm^−1^ and 1261.40–1262.05 cm^−1^, and between healthy children and children with CF in 1260.70 and 1261.65 cm^−1^. While repeatability could be improved, broadband QCL-based spectroscopy is a relatively easy and fast technique.

### 3.7. Brain Cancer

**Figure 9:**
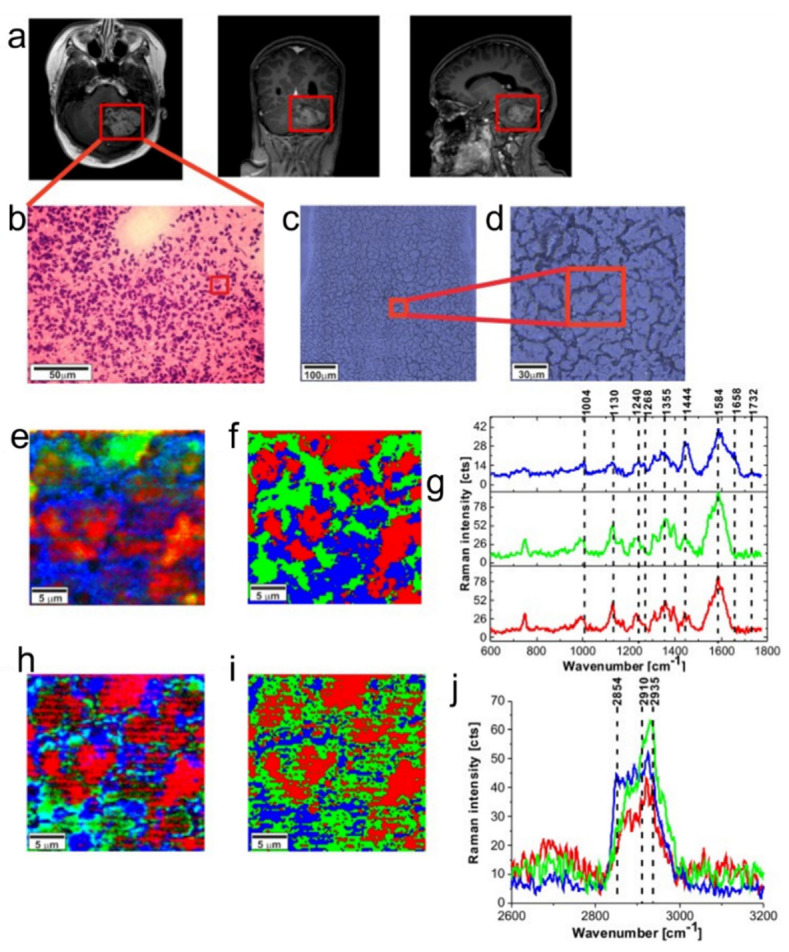
Images and analyses used to study a tumor in the central nervous system. These include an MRI image (**a**), a histological image stained with hematoxylin and eosin (**b**), a microscopy image created by stitching together smaller images (**c**), a microscopy image at higher magnification (**d**), Raman images at a resolution of 50 μm × 50 μm (**e**,**h**), cluster analysis of the Raman data (**f**,**i**), and characteristic vibrational Raman spectra (**g**,**j**) in the high−frequency region. Red is the protein-rich region, blue is the lipid-rich region, and green is the mixed lipid-protein region. (Adapted with permission from Anna et al.) [64].

Anna et al. utilized Raman spectroscopy to compare normal tissue with medulloblastoma, low-grade astrocytoma, ependymoma, and metastatic brain tumors. According to the study, Figure 9 shows that high-grade medulloblastoma samples have reduced levels of saturated fatty acids compared to low-grade astrocytoma and non-tumor brain samples. High-grade brain tumors exhibited significantly lower levels of oleic acid [64]. Tobias Meyer utilized label-free vibrational microspectroscopic imaging as a promising technique for the quick and accurate in vivo diagnosis of brain tumors. The study demonstrated that second harmonic generation (SHG) imaging offered high chemical selectivity, two-photon excited fluorescence (TPEF) allowed for label-free imaging of the morphology, and CARS was promising for imaging the chemical composition [131]. The efficacy of a portable Raman scanner in detecting brain tumors during surgical resection was investigated by Karabeber et al. The findings indicated that the use of a SERS image-guided resection was more precise than relying solely on white light visualization. The hand-held Raman probe successfully identified the microscopic foci of cancer that would have otherwise have gone undetected [132].

Lilo et al. investigated the use of ATR-FTIR spectroscopy and chemometric techniques for distinguishing between different grades of meningioma tumors based on their biospectrochemical profiles [133]. The samples used were FFPE brain tissue samples on slides from 99 patients with meningioma tumors. The results showed that the combined technique could successfully distinguish between grade I, grade II, and grade III meningiomas, with an accuracy of classification of 79% for PLS-DA and 80% for PCA-LDA for discriminating between grade I and grade II meningiomas, while the accuracy was 94% for PLS-DA and 97% for PCA-LDA for discriminating between grade I recurrence and grade II recurrence. The analysis also indicated the spectral fingerprints related to changes in molecular composition associated with different grades of meningioma tumors. These spectral fingerprints were associated with alterations in lipids, proteins, DNA/RNA, and carbohydrates. The study offers a potential non-destructive, low-cost, and sensitive tool for clinical settings to aid in determining the grade and biospectrochemical profiling of meningioma tumors, which could improve patient outcomes and advance our understanding of these types of tumors. 

Hands et al. investigated the potential of ATR-FTIR spectroscopy in discriminating between different levels of brain tumor severity using serum samples [134]. ATR-FTIR spectroscopy was used to analyze the molecular composition of serum samples from patients with different grades of brain tumors. Serum samples from 112 patients with different grades of brain tumors, including 38 low-grade gliomas, 38 high-grade gliomas, and 36 metastatic brain tumors were measured. The authors identified spectral fingerprints related to changes in molecular composition that were associated with different levels of brain tumor severity. Specifically, changes in lipid composition were found to be associated with higher-grade tumors. The sensitivity and specificity for distinguishing between low-grade gliomas and high-grade gliomas were 94% and 97%, respectively, while the sensitivity and specificity for distinguishing between high-grade gliomas and metastatic brain tumors were 100% and 97%, respectively. The study provides a non-invasive method for diagnosing and monitoring brain tumors using serum samples. It shows that ATR-FTIR spectroscopy could potentially be used to improve patient outcomes by enabling earlier detection and more accurate monitoring of tumor progression.

Gajjar et al. investigated the use of ATR-FTIR and Raman spectroscopy combined with multivariate analysis as a potential diagnostic tool for brain tumors [135]. A total of 278 samples were analyzed, which were tissue sections from normal brain tissue, meningioma, glioma, and brain metastases. The spectral data analysis showed changes in the brain’s biochemical structure in relation to different tumor types. Specifically, there was a tentative link between a decrease in the lipid-to-protein ratio and increased tumor progression. Additionally, an alteration in the ratio of cholesterol esters to phenylalanine was visible in metastatic and grade IV glioma tumors. These changes were detected using biospectroscopy techniques with high sensitivity (up to 100%) and specificity (up to 98%). This study has significant implications for the diagnosis and treatment of brain tumors by providing a more objective and precise diagnosis compared to current methods, potentially improving patient outcomes by aiding in surgical planning and treatment decisions.

### 3.8. Thyroid Cancer

O’Dea et al. used a benign thyroid cell line and seven thyroid cancer cell lines to develop a diagnostic algorithm using Raman spectroscopy. The variances in spectra observed between cancer and benign cells were attributed to differences in the composition of nucleic acids, lipids, carbohydrates, and proteins. Raman spectroscopy was accurate in identifying thyroid cancer, with sensitivities ranging from 74% to 85%, specificities from 65% to 93%, and diagnostic accuracy from 71% to 88% [136].

Santillan and colleagues conducted a study on the use of artificial neural networks in predicting thyroid cancer using ATR-FTIR spectroscopy [137]. They identified specific peaks and cluster patterns in the fingerprint IR regions that could effectively distinguish between benign and cancerous thyroid lesions. The samples were thyroid tissues on glass slides obtained from 164 patients with either benign or malignant lesions. The study found that the neural network models designed using ATR-FTIR input data showed high accuracy in diagnosing thyroid malignancy, with an overall accuracy of 94.5%, sensitivity of 93.8%, and specificity of 95.2%. The study presents a method that is more objective and efficient in distinguishing between benign and malignant thyroid tissues by utilizing FTIR spectroscopy and artificial neural networks. This method has potential applications in clinical practice for disease diagnosis and prognosis.

### 3.9. Leukemia

Sheng et al. conducted a study to explore the feasibility of FTIR spectroscopy for distinguishing between serum samples from patients with leukemia and those from healthy individuals [138]. The researchers analyzed a total of 30 serum samples, including 15 from leukemia patients and 15 from healthy individuals. While the IR spectra of both groups showed similarities, specific ratios exhibited notable differences. In particular, leukemia patients had a higher H2964/H2926 ratio but a lower RNA/DNA ratio compared to healthy individuals. The study suggests that FTIR spectroscopy could serve as a promising method for diagnosing leukemia by detecting differences in specific ratios between serum samples from leukemia patients and healthy individuals. However, further research is needed to confirm these findings and determine the full potential of FTIR spectroscopy as a diagnostic tool for leukemia.

### 3.10. Bladder Cancer

Crow et al. investigated the potential of Raman spectroscopy in detecting and grading bladder cancer. Raman spectra were collected from 75 bladder samples, including normal bladder, cystitis, carcinoma in situ, transitional cell carcinoma, and adenocarcinoma. The spectral data were then analyzed using multivariate technologies to create diagnostic algorithms [139]. 

De Jong et al. studied the use of Raman spectroscopy to differentiate between nontumor and tumor bladder tissue. The study also showed that the spectral differences were due to the higher collagen content in nontumor tissue and higher lipid, nucleic acid, protein, and glycogen content in tumor tissue [140]. 

Gao et al. suggested that a mathematical model based on the biochemical characteristics of normal and cancerous tissues needs to be constructed to improve the diagnostic accuracy of Raman spectroscopy [65]. The paper by Auner et al. detailed the potential clinical applications of Raman spectroscopy for cancer detection. They explained how this technique could be utilized to identify different types of cancer [67]. 

An optical fiber probe for real-time in vivo cancer diagnosis was developed by Wang et al. This probe can be utilized for the diagnosis of various types of cancer, including skin, lung, stomach, esophageal, colorectal, cervical, and breast cancer [14]. Khan et al. combined Raman spectroscopy and a support vector machine (SVM) to analyze human serum and identify nasopharyngeal cancer (NPC) [141].

Gok et al. proposed FTIR spectroscopy as a more sensitive, rapid, non-destructive, and operator-independent diagnostic method for bladder cancer recurrence from bladder wash samples [142]. The study recruited 136 patients and compared the results of transmission FTIR and ATR-FTIR spectroscopy with those of urine cytology and cystoscopy. For the transmission FTIR measurements, the samples were in powder form mixed with KBr. For the ATR-FTIR measurements, the samples were liquid bladder wash samples. A statistical analysis and PCA were carried out for classification. The study found significant differences in molecular content between the bladder cancer and control groups using FTIR spectroscopy, with the best discriminations in the 1500–1340 cm^–1^, 1100–900 cm^–1^, and 900–800 cm^–1^ bands. FTIR spectroscopy coupled with chemometrics was able to successfully differentiate the diseased group from the control group with a sensitivity value of 100% for the carcinoma group. The study found that FTIR spectroscopy offers quicker and more reliable results than cytology, and it can enable the early detection of bladder tumors non-invasively and determine the bladder tumor patients that require cystoscopy during the follow-up period.

### 3.11. Ovarian Cancer

Lima et al. identified spectral biomarkers for the accurate diagnosis of ovarian cancer, including the cancer stages, histological type, and age differences using ATR-FTIR and the genetic algorithm or successive projection algorithm combined with LDA [143]. The samples used in the study were plasma or serum specimens from 30 patients with ovarian cancer. The study showed that when using plasma blood, the sensitivity and specificity levels were 100% for segregating stage I vs. stage II-IV, up to 94% for the serious vs. non-serious category, and complete accuracy (100%) was achieved for the 60 years and >60 years categories using the selected wavenumbers. For example, in plasma samples, the variables at 1323 and 1350 cm^−1^ were found to be of particular interest in distinguishing between stage I and stage II-IV, representing the symmetric stretching of the carboxyl groups of amino acid side chains and collagen, respectively. For serum samples, high sensitivity and specificity results were achieved (up to 91.6% stage I vs. stage II–IV; up to 93.0% serious vs. non-serious; and up to 96.0% for 60 years vs. >60 years). This suggests that ATR-FTIR spectroscopy can be used as a screening tool for ovarian cancer.

**Figure 10:**
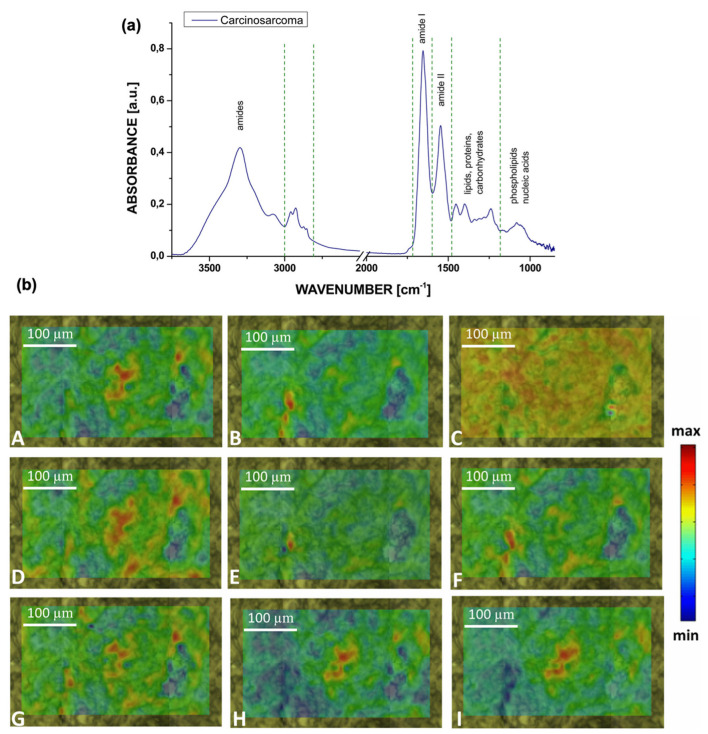
(**a**) FTIR spectrum of the malignant ovarian cancer tissue with the spectral bands marked. (**b**) Spatial distribution maps for the malignant type of tumor obtained from the microscopic view. A: distribution of the proteins, amide I band (1660 cm^−1^), B: distribution of the proteins, amide I band (1553 cm^−1^), C: structural changes of proteins (1660 cm^−1^/1553 cm^−1^), D: distribution of phosphate bond(s) including nucleic acids (1080 cm^−1^), E: distribution of phosphate bond(s) including nucleic acids (1240 cm^−1^), F: protein massif (1700–1500 cm^−1^), G: distribution of lipids (2955 cm^−1^), H: distribution of lipids (2920 cm^−1^), I: distribution of lipids (2850 cm^−1^) Scale bar: 100 μm. (Adapted with permission from Grzelak et al.) [144].

Grzelak et al. utilized synchrotron radiation-based Fourier transform infrared spectroscopy (SR-FTIR) to investigate the molecular composition of ovarian neoplastic tissues based on their biological potential, as presented in Figure 10a [144]. The team placed thin tissue sections on BaF_2_ substrates and analyzed the spatial distribution of various biochemical markers for ovarian tumors, as depicted in Figure 10b. The study examined eight samples of ovarian tissues, and it was revealed that malignant tumors had higher lipid/protein ratios compared to benign tumors. The team employed the mean intensities of biomolecules to distinguish between different tissue types and levels of malignancy in ovarian tumors. The Mann-Whitney U test was used for statistical evaluation, and the results showed significant differences between benign and malignant tumors in terms of amide I intensity ratios (*p* < 0.05) and lipid/protein ratios (*p* < 0.01). The potential benefits of using SR-FTIR spectroscopy in diagnosing ovarian cancer include improved accuracy and efficiency compared to traditional methods (histology), as well as the ability to differentiate between different tissue types and malignancy levels based on the mean intensities of biomolecules.

### 3.12. Biliary Tract Cancer

Untereiner et al. described a pilot study that aimed to determine whether FTIR spectroscopy is able to distinguish bile samples from patients with and without malignant biliary strictures [145]. FTIR spectroscopy was used as a new diagnostic tool for differentiating patients with malignant bile duct strictures from those with benign biliary diseases. A total of 57 bile samples were collected during endoscopic procedures (38 with benign biliary diseases and 19 with malignant diseases). The spectral analysis revealed significant differences between the spectra of malignant and benign bile samples, which were attributed to changes in the molecular composition. For example, the intensity ratio of the lipid carbonyl C=O vibration to the amide I band of proteins (I_1742/1669_) was 1.64 in the bile organic phase spectrum compared to 0.22 in the bile aqueous phase spectrum. The results showed that FTIR spectroscopy could be used to differentiate between biliary tract cancer and benign biliary diseases, with an accuracy rate of 89%. The study demonstrates the potential of FTIR spectroscopy as a non-invasive diagnostic tool for detecting biliary tract cancer, which could lead to earlier diagnosis and improved patient outcomes. However, further studies on a larger population are required to evaluate the potential of this classifier for differentiating between biliary and pancreatic cancers.

### 3.13. Ewing Sarcoma Cancer

Chaber et al. concluded that spectral data obtained from paraffined and deparaffinized bone tissue samples were not significantly different, and the deparaffinization process was not required before spectroscopy [146]. The samples used were bone specimens, where 20 samples were obtained from 10 Ewing sarcoma patients. This study aimed to verify if the FTIR spectrum could detect Ewing sarcoma without deparaffinization. Bone tissue samples were embedded in paraffin, and some sections were deparaffinized before the tissue samples were sliced to produce slides. The ATR-FTIR technique was used for spectral measurement, and paraffin was used as the background for paraffined samples. Gaussian, Lorentz, statistical and computational analyses was performed on the original spectrum data. The analysis conducted on the data found that the peak at 1234 cm^−1^ showed some statistically significant differences in the absorbance, surface area, and half-width of the peak. The analysis using an imaginary dielectric function confirmed that the Lorentz function parameters for individual peaks were the same and did not depend on the sample preparation methods. Hence, deparaffinization does not have to be performed on samples before the spectroscopy results are acquired.

### 3.14. Kidney Cancer

Bogomolov et al. demonstrated the advantages of combining fluorescence and MIR fiber spectroscopy to diagnose kidney tumors through the synergy effect [147]. In this study, eight cryo biopsies were collected from four patients after nephrectomy, with each kidney providing four pairs of non-tumor and tumor tissues. The researchers utilized a polycrystalline infrared (PIR) fiber-based ATR probe with a mercury-cadmium-telluride (MCT) detector for MIR spectroscopy measurements. Fluorescence spectroscopy measurements were conducted using a needle-shaped probe consisting of an aluminum-coated 400 μm core detection fiber and 13 silica illumination fibers of 100 μm in diameter, in addition to a 25 mW laser. The collected data was analyzed using PCA and PLS-DA, which showed that combining the two spectroscopy techniques had a synergistic effect on kidney tumor diagnosis. The accuracy of the combined method was found to be higher than that of the individual spectroscopy methods in calibration, cross-validation, and random-subset validation.

### 3.15. Multiple Cancers

Paraskevaidi et al. recognized the need for an affordable and non-invasive screening and diagnostic test for gynecological cancers [148]. Spectroscopic techniques have demonstrated their potential in disease investigation and diagnosis. To this end, they employed ATR-FTIR spectroscopy to examine urine samples from women with endometrial and ovarian cancer, in addition to healthy individuals, with 10 samples from each group. The researchers analyzed the spectral features associated with proteins, lipids, and nucleic acids, and discussed their findings. For instance, in comparing ovarian cancer patients to healthy controls, they found that the main differentiating factors were peaks related to proteins and nucleic acids. These biomolecules were present in higher concentrations in cancerous samples, except for a peak at 1597 cm^−1^ that corresponds to the C-C phenyl ring of proteins. By using multiple machine learning models, the researchers achieved high levels of accuracy and sensitivity for both types of cancer. This cost-effective and non-destructive method holds promise as a potential diagnostic tool for endometrial and ovarian cancers. Urine collection and subsequent analysis is quick and non-invasive, making it ideal for repeated measurements to monitor disease progression or therapeutic response. A larger study is needed to validate these preliminary results.

**Figure 11:**
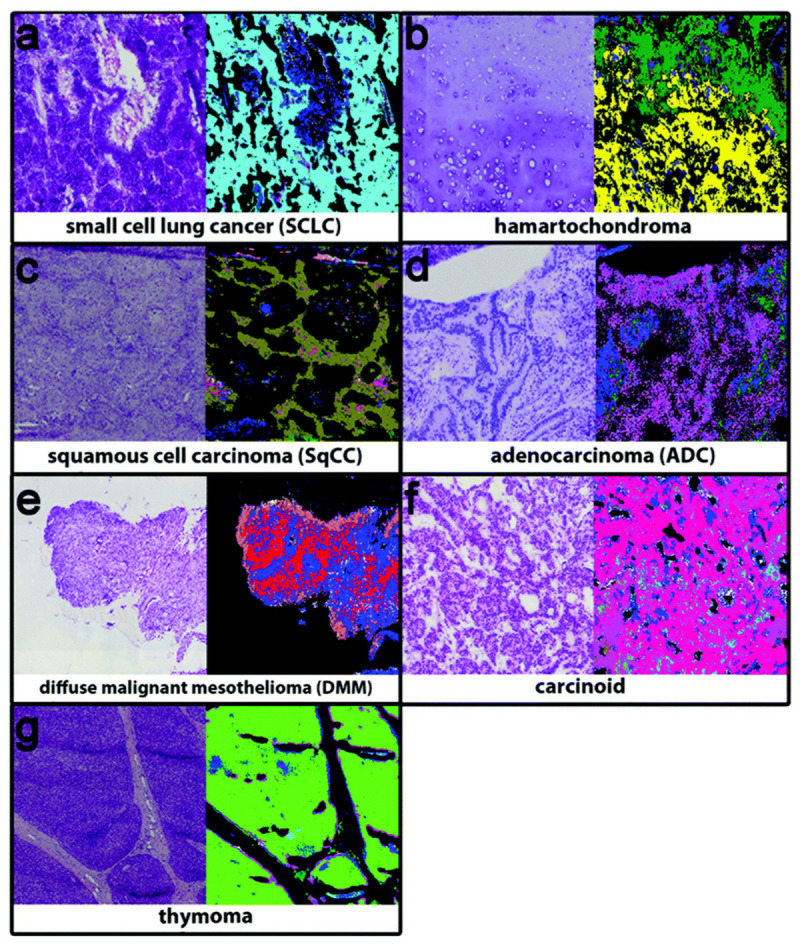
The second level tumor classification. (**a**) Small cell cancer (cyan), (**b**) hamartochondroma (yellow), (**c**) squamous cell carcinoma (olive), (**d**) carcinoid (magenta), (**e**) pleura mesothelioma (red), (**f**) adenocarcinoma (pink), and (**g**) thymoma (light green), and inflammation/necrosis (blue and dark green). (Adapted with permission from Großerueschkamp et al.) [149].

Großerueschkamp et al. proposed a new diagnostic tool that integrates FTIR imaging and a trained random forest classifier to identify lung tumor classes and subtypes of adenocarcinoma in fresh-frozen tissue slices mounted on LowE slides, without the need for markers [149]. A total of 101 patients were analyzed. The decision tree classified healthy and pathologically relevant regions in the first level (healthy/pathologic), five tumor classes in the second level (pathological classification), and subtypes for each tumor class in the third level (subtypes of adenocarcinoma), as shown in Figure 11. The authors highlighted that this is a significant achievement, as they were able to differentiate not only between different types of cancer with a high level of accuracy (97%), but also between different subtypes of adenocarcinoma with an accuracy of 95%. The method is more accurate and reproducible compared to previous studies, and the differentiation of subtypes is important for the prognosis and therapeutic decision. This approach has the potential to reduce variability and improve accuracy in lung tumor diagnostics for personalized medicine, and future validation is required.

Menzies et al. explored the possibility of using FTIR spectroscopy as an affordable and non-invasive diagnostic tool to identify head and neck cancer in its early stages [150]. The authors collected sputum samples from patients with oral and oropharyngeal (16 cases), laryngeal cancer (eight cases), as well as from normal controls (15 cases), and used FTIR to generate spectra in the biochemical fingerprint region. The study found that FTIR was able to discriminate between cancer and normal sputum using the infrared wavenumbers 1650 cm^−1^, 1550 cm^−1^, and 1042 cm^−1^, and that the method had the potential to detect laryngeal tumors that are hidden from noninvasive observation.

**Figure 12:**
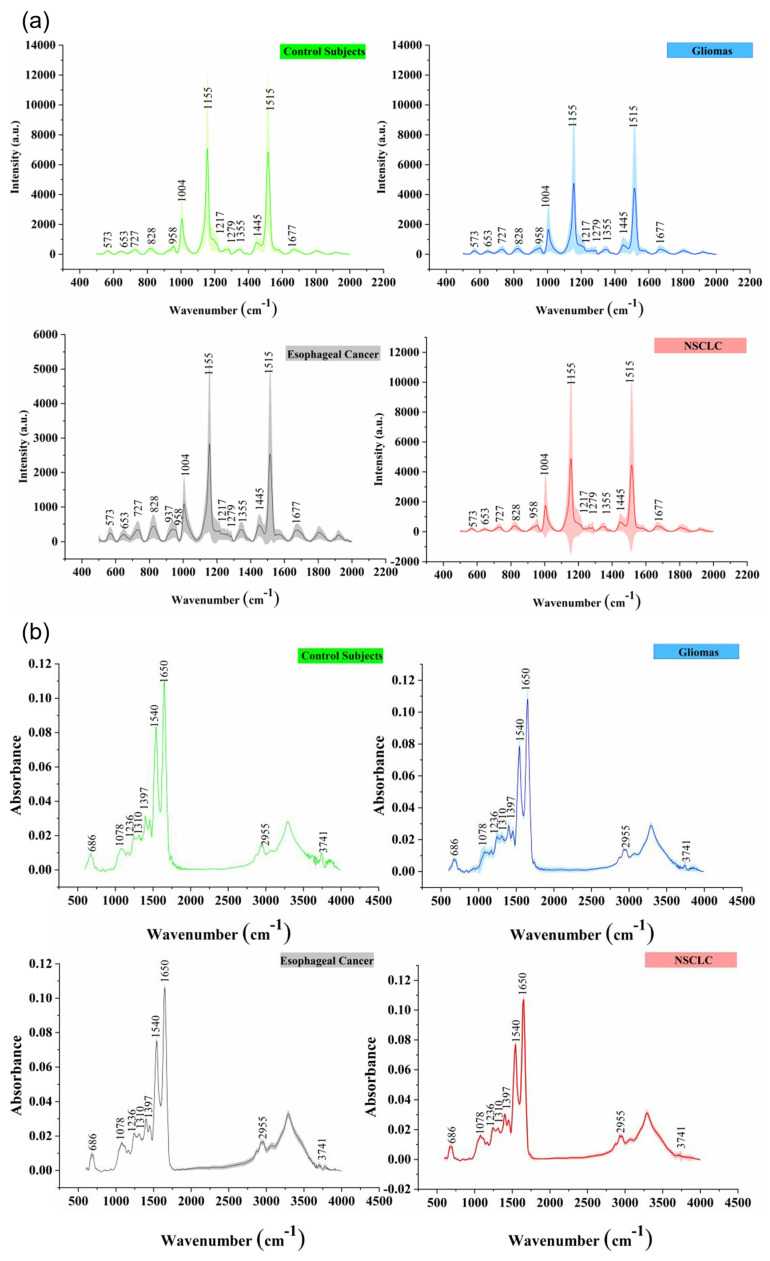
Measurement results of different classes using (**a**) Raman spectroscopy and (**b**) FTIR spectroscopy. (Adapted with permission from H. Leng, et al.) [151].

Leng et al. proved that the fusion technology of spectroscopy data and deep learning for cancer prediction resulted in better performance than single spectral data [151]. A total of 164 blood samples were collected, with 45 from the control group, 44 from non-small cell lung cancer (NSCLC) patients, 38 from glioma patients, and 37 from esophageal cancer patients. These samples were measured using two spectroscopy methods: FTIR and Raman, as shown in Figure 12. The spectrum from these two methods was then fused using low-level and feature fusion. Several classification models based on deep learning, such as SVM, convolutional neural network-long-short term memory (CNN-LSTM), and multi-scale fusion convolutional neural networks (MFCNN) were created and evaluated. Each model was trained using single spectral data—Raman and FTIR, as well as low-level fused spectral data and feature-fused data. The highest overall accuracy achieved was 82.51%, from the CNN-LSTM model using low-level fusion data. The authors found that the accuracy of these fused models resulted in a 10% increase compared to single spectral models. 

## 4. Conclusions

Molecular fingerprint detection using the Raman and infrared spectroscopy technologies has become increasingly popular for biomedical applications. These technologies provide detailed information about the chemical composition and structure of biological samples, such as tissues, cells, and fluids. Both technologies provide unique “fingerprints” of the molecules in the sample which can be used to identify the chemical bonds, functional groups, and structures of the molecules.

In this article, we first reviewed both technologies in detail, including the different modifications to improve sensitivity and functionality, after which we surveyed a variety of biomedical applications which employed either one technique or a combination of both and discussed the important findings. Table 3 is a list of abbreviations. Raman spectroscopy is an invaluable tool for studying molecules and their interactions, and its use is likely to continue to grow in the future. Research has demonstrated that Raman spectroscopy can accurately diagnose various types of cancer, including gastric, bladder, colon, oral, prostate, breast, ovarian, and cervical cancers. The method is non-invasive, real-time, and requires no additional reagents, making it a valuable alternative to traditional diagnostic methods such as endoscopy. Raman spectroscopy is generally more sensitive to bonds involving heavy atoms such as C-H, C-N, and C-O bonds, while infrared spectroscopy is more sensitive to bonds involving light atoms, such as C-C, C-H, and O-H bonds. This difference in sensitivity can affect the ability of the technologies to provide information about the functional groups and the bonds of the molecules in a sample. In addition, Raman spectroscopy can suffer from autofluorescence signals, and infrared spectroscopy can suffer from water interferences. Hence, we have seen a trend whereby multimodality measurements provide a complete characterization of the sample and generally yield high sensitivity and accuracy in medical diagnoses. 

The use of multivariate data analysis technologies, such as partial least squares regression and partial least square discriminant analysis, further improves the accuracy of the diagnostic results. The results have shown that Raman and infrared spectroscopy combined with multivariate data analysis can be used as a reliable and efficient tool for health screening. However, it is also important to consider the limitations. While these methods have demonstrated promising results in distinguishing between healthy and diseased samples, it is not accurate to claim that they can diagnose a specific disease. Binary classification between healthy and diseased states is typically used, and can be affected by physiological conditions and medical treatments. It is therefore important to carefully interpret the results of these studies and to consider the potential confounding factors. Future research should aim to explore the ability of Raman and IR spectroscopy to distinguish between different types of cancers and to identify specific disease patterns, while controlling for the effects of medical treatment.

Overall, vibrational spectroscopy involving Raman and infrared spectroscopy is a valuable tool for molecular fingerprint detection, and can provide important information about the chemical composition and structure of biological samples. The choice of which technique to use will depend on the specific needs and goals of the research, as well as the type of molecules and functional groups that are of interest. Finally, technological developments could lead to improvements in the speed, portability, and cost of the systems, because these are the determinant factors for the likelihood of technology adoption in a future clinical setting.

## Figures and Tables

**Table 1:** Characteristic peaks for prostate cancer using SERS. Adapted with permission from Harron et al. [71].

Peaks (cm^−1^)	Assignment	Remarks	Substrate
484	C-C str	Glycogen molecule	AuNPs
492		glycogen	AuNPs
495		Uric acid	AuNPs
529	S-S	protein	AuNPs
532	Zn2+	Zinc ion	AuNPs
619		Xanthene ring	AuNPs
719–726		DNA/RNA	
727		Hypoxanthine	
797	O-P-O	DNA	
887.68	C-O-H		
935–937	C-C str	Protein	
960		Carotenoid	
1002		Phenylalanine	
1062	C-C	Lipid	
1087	P-O	Phosphoproteins	
1134		D-Mannose	
1155	C-C, C-N str	Proteins	
1160		PSA	
1171	C-H str	Protein	
1326	N=O str		AuNPs
1356		RhodamineB	AuNPs
1426		Creatine	
1490	NH3 str	Glutamine	
1523		Carotenoids	

**Table 2:** Major vibrational bands of the colorectal cancer serum samples. (Adapted with permission from Lin et al. [99]).

Peak Positions (cm^−1^)	Vibrational Mode	Major Assignment
494	ν(S-S)	L−arginine
589		Amide−VI
638	ν(C-S)	Tyrosine
725	δ(C-H)	Adenine
823	Ring breathing	Tyrosine
881	δ(ring)	Tryptophan
1004	νs(C-C)	Phenylalanine
1074	ν(C-C)	Phospholipids
1206	Ring vibration	Tyrosine
1322	CH_3_CH_2_ twisting	Collagen, tryptophan
1365		Tryptophan
1655	ν(C=O)	Amide I

**Table 3:** List of abbreviations.

Attenuated total reflection	ATR
Attenuated total reflection surface-enhanced infrared absorption spectroscopy	ATR-SEIRAS
Basal cell carcinoma	BCC
Chalcogenide infrared	CIR
Coherent anti-Stokes Raman spectroscopy	CARS
Colorectal cancer	CRC
Convolutional neural network-long-short term memory	CNN-LSTM
Cystic fibrosis	CF
Deuterated triglycine sulfate	DTGS
Electrochemical-SERS	EC-SERS
Electromagnetic	EM
Extreme learning machine	ELM
Femtosecond stimulated Raman spectroscopy	FSRS
Focal plane array	FPA
Formalin-fixed and paraffin embedded	FFPE
Fourier transform	FT
Fourier transform infrared	FTIR
Gastrointestinal	GI
Hepatitis B virus	HBV
High-risk	HR
Immunoglobulin	IgG
Invasive ductal carcinoma	IDC
Laser absorption spectroscopy	LAS
Leave-one-out-cross-validation	LOOCV
Linear discriminant analysis	LDA
Localized surface plasmon resonance	LSPR
Low-risk	LR
Machine learning	ML
Mercury-cadmium-telluride	MCT
Mid-infrared	MIR
Multi-scale fusion convolutional neural networks	MFCNN
Nasopharyngeal cancer	NPC
Near-infrared	NIR
Non-small cell lung cancer	NSCLC
Octadecanethiol	ODT
Partial least squares discriminant analysis	PLS-DA
Point-of-care	POC
Polycrystalline infrared	PIR
Principal component analysis	PCA
Principal component regression	PCR
Quantum cascade laser	QCL
Radial basis function	RBF
Resonance Raman spectroscopy	RRS
Root mean square error	RMSE
Second harmonic generation	SHG
Signal-to-noise ratio	SNR
Single nucleotide polymorphism	SNP
Small cell lung carcinoma	SCLC
Spatially offset Raman spectroscopy	SORS
Stimulated Raman spectroscopy	SRS
Support vector machine	SVM
Surface-enhanced infrared absorption	SEIRA
Surface-enhanced Raman spectroscopy	SERS
Synchrotron radiation-based FTIR	SR-FTIR
Tetrahedral DNA nanostructure	TDN
Tip-enhanced Raman spectroscopy	TERS
Two-photon excited fluorescence	TPEF
Ultraviolet	UV
Urinary extracellular vesicles	UEV
Vertically coupled complementary antennas	VCCA
Visible	VIS
Volatile organic compounds	VOC

## Data Availability

No new data was created.
